# A Machine‐Learning Approach Identifies Rejuvenating Interventions in the Human Brain

**DOI:** 10.1002/advs.202503344

**Published:** 2025-07-14

**Authors:** Guillem Santamaria, Cristina Iglesias, Sascha Jung, Javier Arcos Hodar, Ruben Nogueiras, Antonio del Sol

**Affiliations:** ^1^ Luxembourg Centre for Systems Biomedicine (LCSB) University of Luxembourg 6 Avenue du Swing Esch‐Belval Esch‐sur‐Alzette 4367 Luxembourg; ^2^ CIC bioGUNE‐BRTA (Basque Research and Technology Alliance) Bizkaia Technology Park 801 Building Derio 48160 Spain; ^3^ IKERBASQUE Basque Foundation for Science Bilbao 48013 Spain; ^4^ Centro de Investigación en Medicina Molecular y Enfermedades Crónicas (CIMUS) University of Santiago de Compostela (USC) Instituto de Investigación Sanitaria (IDIS) Campus Vida Avenida Barcelona s/n Santiago de Compostela 15782 Spain

**Keywords:** cognitive decline, gene expression, senescence, therapies, transcriptional age

## Abstract

The increase in life expectancy has caused a rise in age‐related brain disorders. Although brain rejuvenation is a promising strategy to counteract brain functional decline, systematic discovery methods for efficient interventions are lacking. A computational platform based on a transcriptional brain aging clock capable of detecting age‐ and neurodegeneration‐related changes is developed. Applied to neurodegeneration‐positive samples, it reveals that neurodegenerative disease presence and severity significantly increase predicted age. By screening 43840 transcriptional profiles of chemical and genetic perturbations, it identifies 453 unique rejuvenating interventions, several of which are known to extend lifespan in animal models. Additionally, the identified interventions include drugs already used to treat neurological disorders, Alzheimer's disease among them. A combination of compounds predicted by the platform reduced anxiety, improved memory, and rejuvenated the brain cortex transcriptome in aged mice. These results demonstrate the platform's ability to identify brain‐rejuvenating interventions, offering potential treatments for neurodegenerative diseases.

## Graphical abstract

Rising life expectancy increases the incidence of age‐related neurodegeneration. Reverting brain aging through effective rejuvenation strategies constitutes a promising strategy to counteract these disorders and restore brain function. This study uses a brain‐specific transcriptional aging clock to screen over 43 000 perturbation profiles, identifying novel interventions. A predicted perturbation combination reduces anxiety, improves memory, and rejuvenates the cortical transcriptome in aged mice.

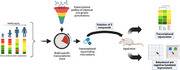

## 1 Introduction

The global population is aging rapidly, with over two billion people projected to be above the age of 60 by 2050.^[^ ^1^ ^]^ While increased lifespan is positive, it has led to a rise of age‐related conditions, resulting in more individuals living longer in poor health.^[^ ^2^, ^3^ ^]^ In 2017, age‐related diseases accounted for more than 50% of the global disease burden and are expected to rise further.^[^ ^4^ ^]^ This poses significant health challenges and places substantial financial pressure on healthcare systems. Neurodegenerative disorders comprise a large portion of the disease burden.^[^ ^5^ ^]^ Therefore, discovering effective strategies to protect the aging population from neurodegeneration is critical.

Neurodegeneration and aging are closely interconnected. During aging, cellular homeostasis declines, leading to increased neuroinflammation, DNA damage, oxidative stress, and mitochondrial malfunction. Over time, quality control systems fail to counteract these defects, eventually leading to neuropathological states.^[^ ^6^, ^7^, ^8^ ^]^ As such, aging is the primary risk factor for several neurodegenerative disorders that most older adults eventually face. While it remains debated whether neurodegeneration represents accelerated aging or a distinct pathological process,^[^ ^9^ ^]^ there is growing evidence of shared molecular hallmarks that are exacerbated in patients.^[^ ^10^, ^11^, ^12^ ^]^

The concept of neurodegeneration as accelerated aging has spurred interest in rejuvenation‐based interventions for treatment.^[^ ^12^, ^13^, ^14^ ^]^ Promising approaches as parabiosis, exercise and caloric restriction, have shown positive results in slowing and reversing neurodegeneration.^[^ ^14^, ^15^, ^16^, ^17^, ^18^, ^19^ ^]^ Chemical rejuvenating interventions like senolytics and SIRT1 activators (e.g., resveratrol) also have been shown to mitigate neurodegenerative traits.^[^ ^20^, ^21^ ^]^ At the molecular level, these interventions reprogram the transcriptional state of cells and restore their methylome to a more youthful profile.^[^ ^22^, ^23^, ^24^, ^25^, ^26^ ^]^ Furthermore, a recent study showed that rejuvenating the mouse brain by inducing Yamanaka factors expression prevented the development of several hallmarks of Alzheimer's disease.^[^ ^27^ ^]^ However, these strategies present notable limitations. For example, resveratrol suffers from low bioavailability, and interventions that promote cellular de‐differentiation raise safety concerns.^[^ ^28^, ^29^ ^]^ In addition, the application of parabiosis to humans involves complex ethical issues.

Current interventions have been identified through knowledge‐based targeting strategies. A systematic identification of rejuvenating interventions for the central nervous system would be highly beneficial. The observation that various omics profiles are reprogrammed after applying brain rejuvenating interventions highlights the potential of using omics aging clocks to identify interventions that possess rejuvenating effects.^[^ ^30^ ^]^ Methylation clocks have been regarded as the best given the stability of methylation compared to other omics and their accuracy for estimating age, as evidenced per multiple studies.^[^ ^31^, ^32^ ^]^ However, epigenetic clocks are less functionally interpretable compared to other omics approaches, such as transcriptomics and proteomics, which provide direct insights into altered cellular functions.^[^ ^33^ ^]^ Furthermore, methylation changes take longer to manifest. Another limitation is that most clocks are based on peripheral tissues, which may not capture the brain‐specific changes. While multi‐tissue epigenetic clocks have shown positive correlation with neurodegeneration traits,^[^ ^34^ ^]^ brain‐specific clocks demonstrate stronger associations with neuropathological traits.^[^ ^35^, ^36^ ^]^ Lastly, the use of transcriptomics over epigenomics offers the advantage of being more cost‐effective, facilitating its systematic application in computational drug screening pipelines. However, this comes at the cost of greater variability, making it more challenging to achieve the level of accuracy typically observed with epigenetic clocks.

**Figure 1:**
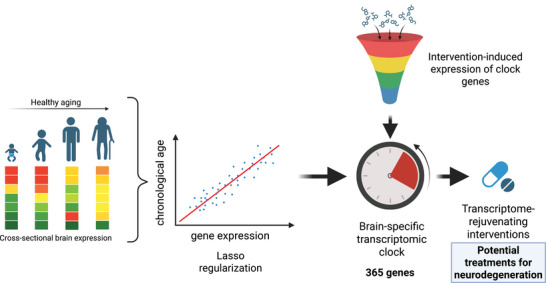
Overview of the development and use of our computational platform for identifying brain rejuvenating interventions. At the core of our platform is a brain‐specific transcriptomic clock, trained on cross‐sectional bulk brain expression data from healthy individuals, aged 20 to 97. This clock employs a generalized linear model, with log2 quantile‐normalized counts as explanatory variables and chronological age as the response variable. Through Lasso regularization, a set of 365 key predictors of brain age was identified. By applying our platform to pre‐ and post‐treatment gene expression data, the clock can identify interventions that significantly rejuvenate the transcriptome. Given the demonstrated positive association between neurodegeneration and transcriptional age, we hypothesize that interventions predicted to reduce transcriptional age may also serve as neuroprotective agents against neurodegenerative disorders.

Recently, we developed a functionally interpretable cell type‐specific transcriptional aging clock that identifies rejuvenating factors in human skin fibroblasts.^[^ ^37^ ^]^ However, the limited availability of brain cell type‐specific gene expression data throughout human lifespan hinders the development of brain cell type‐specific transcriptomic clocks. Nevertheless, a tissue‐specific clock can serve as a suitable proxy for identifying rejuvenating perturbations in brain cell types. In this study, we evaluated 43 840 transcriptional profiles of 5771 chemical and genetic perturbations in neural progenitor cells (NPCs) and neurons for their rejuvenating properties. To achieve that, we developed a computational platform that identifies interventions that significantly shift the transcriptional age of brain cells, based on a transcriptomic brain‐specific aging clock that accurately predicts age from a signature of 365 genes (**Figure** **1**). Our platform identified 971 unique perturbations that rejuvenated the transcriptome in NPCs, and 68 in neurons, many of which had been previously validated. The positive association between neurodegeneration severity and transcriptional age suggests that the interventions predicted as rejuvenating could serve as neuroprotective agents against neurodegenerative disorders. Finally, we tested three of the identified compounds on aged mice, observing reduced anxiety and improved memory, and transcriptomic rejuvenation of the cortical transcriptome. Thus, we anticipate that our computational platform, implemented as the R package 'brainAgeShiftR', will serve as a valuable resource for identifying compounds with therapeutic potential in neurodegenerative diseases, providing a foundation for further research and validation.

## 2 Results

### 2.1 Our Computational Platform can Accurately Predict the Chronological Age of Brain Bulk Transcriptomic Profiles, Identifying Brain‐Specific Aging Signatures

In order to evaluate the rejuvenating properties of chemical and genetic perturbations, we developed a computational platform using bulk transcriptome data from brain samples. Compared to the epigenome, the transcriptome offers the advantage of being highly interpretable, thus allowing for a direct mapping of genes to specific functions and enabling the identification of age‐related processes in the brain. Importantly, we employed bulk data originating from the whole tissue instead of single‐cell data. The motivation behind this choice was twofold, first, the greater availability of brain bulk samples in humans, driven by its lower costs and earlier adoption, second, the fact that drug screenings for the discovery of novel compounds are initially performed on cell cultures, from which bulk transcriptomic profiles can be easily obtained.

We used a combination of bulk RNA‐seq datasets sourced from the Alzheimer's Disease (AMP‐AD) Knowledge Portal,^[^ ^38^ ^]^ the Genotype‐Tissue Expression (GTEx) project,^[^ ^39^ ^]^ the Aging, Dementia and Traumatic Brain Injury Study (TBI),^[^ ^40^ ^]^ and the BrainSeq Phase 2 study^[^ ^41^ ^]^ to train the machine‐learning model underlying our platform, a brain‐specific transcriptional aging clock. From the combination of these datasets, we obtained a total of 2456 samples coming from 778 unique healthy individuals, ranging from ages 20 to 97 years. After data preprocessing (including normalization, transformation, and batch effects removal – see Experimental section for details), we split the samples into training and testing sets (2, 1), stratifying by donor to avoid data leakage. A generalized linear model was then fit on the training set, using the chronological age as the response variable. The expression of all the genes common to all included datasets was used as explanatory variables, with L1 regularization applied to determine what genes significantly contribute to age explanation.

**Figure 2:**
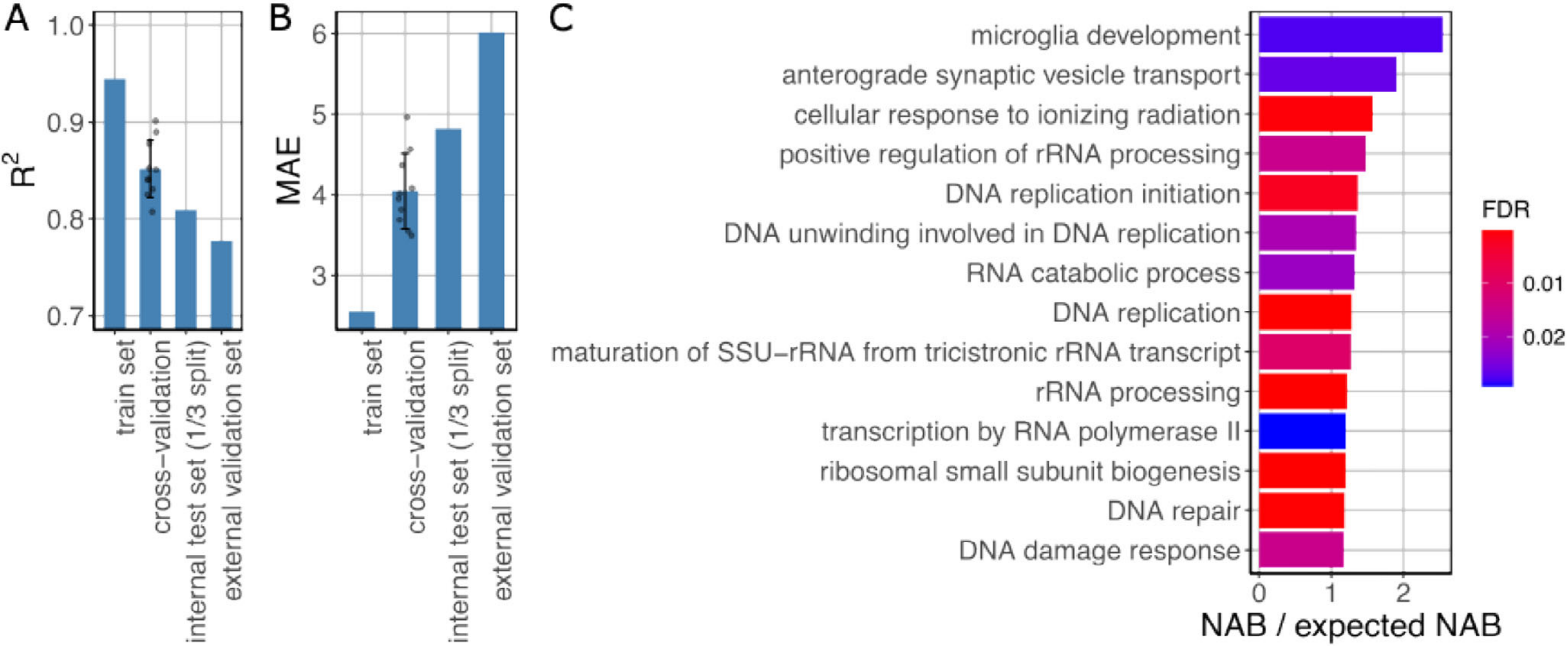
Performance metrics of our transcriptome‐based brain aging clock and main biological processes the genes used by it are involved in. A) R^2^ computed in training set, 10‐fold cross‐validation, internal test set (1/3 held out), and external validation set. B) Mean average error computed in train set, 10‐fold cross‐validation, internal test set (1/3 held out), and external validation set. C) Enriched biological processes in the expression signatures of brain aging used by our clock, determined by NEAT enrichment, and sorted according to FDR. In the X axis it is represented the ratio between the number of links between the genes used by our clock and the biological process and the expected number of links in the absence of enrichment is represented.

The final clock uses 365 genes to make predictions (Table S1, Supporting Information), achieving a coefficient of determination (R^2^) of 0.945 in the training set, an average R^2^ of 0.857 for 10‐fold cross‐validation, and R^2^ 0.808 in the testing set (**Figure** **2**A, **Table** **1**). The mean absolute error (MAE) was 2.55 years in the training set, an average of 4.04 years in 10‐fold cross‐validation, and 4.81 years in the test set (Figure 2B, Table 1). Other metrics for the testing set are shown in Table 1.

**Table 1:** Performance metrics of the transcriptomic clock on the internal (1/3 held‐out) test set and the external validation set.

Metric	Internal test set	External validation set
MAE	4.814	6.013
MSE	37.251	51.422
RMSE	6.103	7.171
MAPE	9.208	13.516
R^2^	0.808	0.778
Pearson^2^	0.813	0.865

Additionally, to assess the clock's performance across the different sub‐studies, brain regions, and sexes represented in our dataset, we computed both R^2^ and MAE for each of these subsets, extracted from the internal test set (Figure S1, Supporting Information). While R^2^ dropped in certain datasets — such as MSBB, TBI or, to a lesser extent, ROSMAP — this was largely due to the limited age range within those cohorts, often spanning no more than two decades (Figure S1A,B, Supporting Information). In such cases, R^2^ becomes a less reliable indicator of model fit. Nevertheless, the MAE across all datasets remained between 3.91 and 8.55 years, indicating acceptable prediction accuracy (Figure S1C, Supporting Information). When stratifying by sex, R^2^ was 0.844 in females, and 0.780 in males (Figure S1E, Supporting Information). This modest decrease in male performance might be attributable to a narrower distribution of chronological ages, likely due to lower male longevity (Figure S1D, Supporting Information). MAE was nearly identical between sexes, 4.82 years in males and 4.8 in females (Figure S1F, Supporting Information).

Performance across individual brain regions followed a similar pattern to the sub‐study analysis, R^2^ values were positively correlated with the standard deviation of chronological ages (Figure S1G, Supporting Information). MAE across regions ranged from 3.695 and 6.685 years (Figure S1H, Supporting Information), supporting the model's accuracy across anatomical contexts.

Finally, we evaluated the model on a completely independent external validation set — 22 samples from the BrainSeq Phase 1 study^[^ ^42^ ^]^ — achieving an R^2^ of 0.778 and a MAE of 6.01 years (Figure 2A,B, Table 1). Altogether, these results show our clock's strong predictive performance and generalizability across diverse biological and technical conditions.

The 365 genes constitute the expression signatures of brain aging. Among these genes, 91 are directly related to brain processes (Table S2, Supporting Information). The brain‐related GO term with most genes associated to was “synapse”. In order to characterize the 365 genes, we performed a Network Enrichment Analysis Test (NEAT)^[^ ^43^ ^]^ to determine which are the functional implications of these changes in expression (Figure 2C; Figures S2–S4, Supporting Information). We detected 14 significantly enriched biological processes (Table S3 and Figure S2, Supporting Information), 11 molecular functions (Table S4 and Figure S3, Supporting Information), and 12 cellular components (Table S5 and Figure S4, Supporting Information). The process with highest ratio of enrichment was microglia development. Interestingly, the majority of significantly altered processes were not directly related with brain pathology. Instead, most of them were associated with DNA metabolism and repair (Figure 1C; Figure S2, Supporting Information). Unexpectedly, the enriched cellular components were structures related to DNA as well, such as nucleoplasm, chromosomes, and nucleolus (Figures S4 and S5B, Supporting Information). Some lysosome‐related components appeared too. A similar trend is observed in molecular functions, where many of the enriched terms are DNA‐related (Figures S3–S5A, Supporting Information). Interestingly, the most enriched molecular function was sterol transporter activity. Alterations in sterol metabolism have been observed during brain aging.^[^ ^44^ ^]^

### 2.2 A Bulk Transcriptome‐Based Clock is a Good Proxy for Identifying Differences in Transcriptional Age at the Cell Type Level

**Figure 3:**
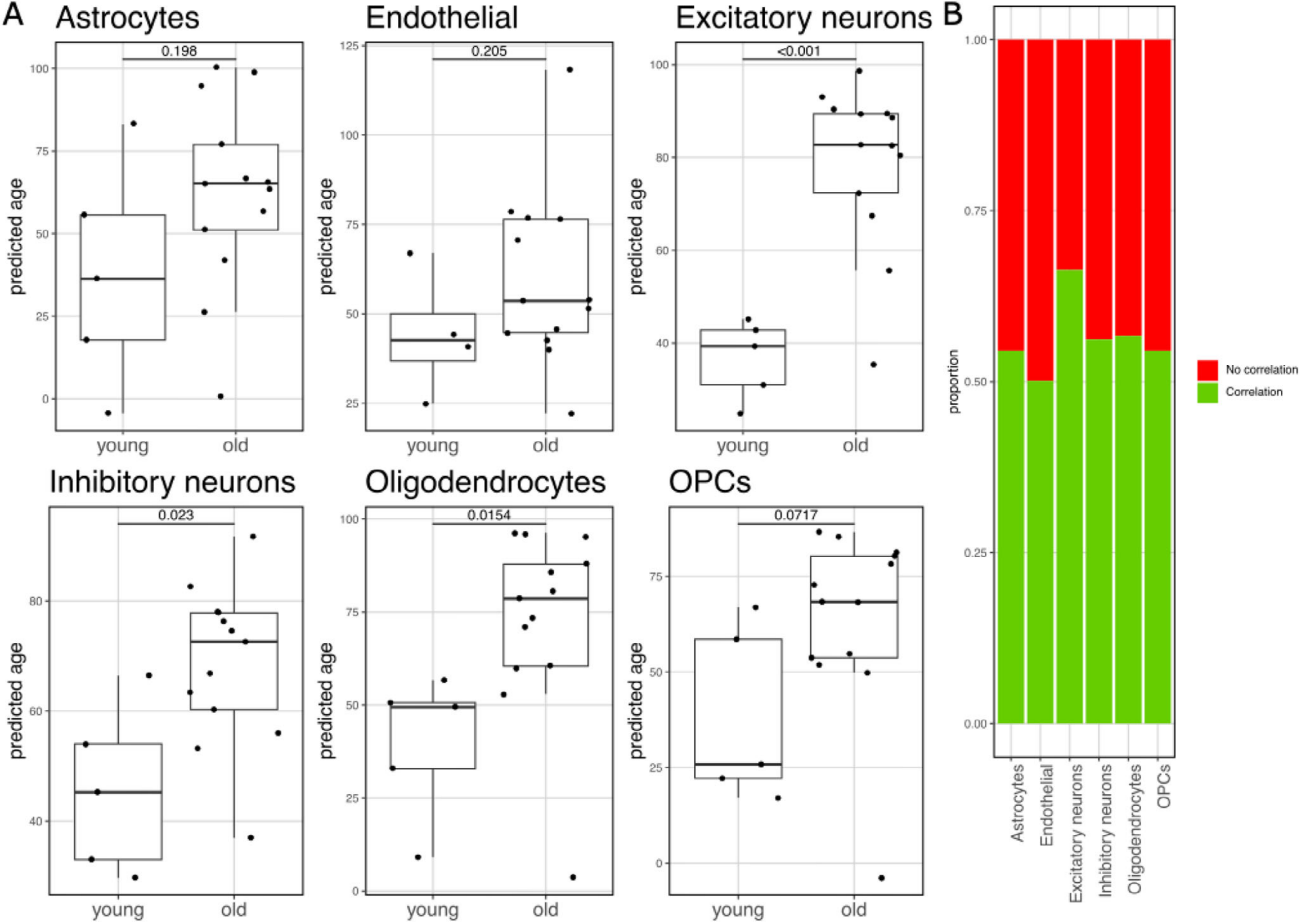
Application of the brain‐specific clock on cell‐type pseudo‐bulk transcriptomic data. A) Comparison of predicted ages per cell type, old (age higher than 70, included) versus young (ages from 18 to 30, both included). The results of a t‐test are displayed on top of each comparison. B) Proportion of genes used by the clock that, in the pseudo‐bulk old versus young individual cell type comparison, have a positive correlation with the coefficient in the clock.

Single‐cell RNA sequencing (scRNA‐seq) has only been widely adopted for about a decade, and its current cost is roughly 10 times higher than bulk RNA‐seq. As a result, there are insufficient scRNA‐seq data of human brain that include the age information necessary to train a high‐quality predictive aging clock for specific brain cell types. However, we used the scRNA‐seq brain datasets available in the ageAnno database^[^ ^45^ ^]^ to validate our clock's ability to capture transcriptional age differences in individual brain cell types. Specifically, we used the preprocessed pseudo‐bulk counts for each individual cell type within each brain sample in the database to predict the ages. We assessed the statistical significance of the differences in the predicted ages between the young (age between 18 and 30) and the old samples (age between 70 and 100) (**Figure** **3**A).

An increase in the median predicted age was observed in old samples across all cell types. Statistically significant differences were found for excitatory neurons, inhibitory neurons and oligodendrocytes, with p‐values of 6.13·10^−6^, 2.3·10^−2^ and 1.5·10^−2^, respectively (3.67·10^−5^, 4.6·10^−2^, and 4.6·10^−2^ after Benjamini‐Hochberg p‐value adjustment). We also examined the proportion of the genes used by the clock that show a positive correlation with the clock's coefficients in a comparison of old versus young samples for each cell type (Figure 3B). These proportions were 0.545 for astrocytes, 0.501 for endothelial cells, 0.663 for excitatory neurons, 0.562 for inhibitory neurons, 0.567 for oligodendrocytes, and 0.545 for OPCs. A permutation test (n = 10000) showed significant enrichment of correlated genes in excitatory neurons, inhibitory neurons, and oligodendrocytes, with BH‐adjusted p‐values of < 1·10^−4^, 2.42·10^−2^, and 2.42·10^−2^, respectively. Finally, F‐tests showed that there is a significant association between the chronological age and the transcriptional age for all the cell types, with the exception of astrocytes (**Table** **2**, Figure S6, Supporting Information).

**Table 2:** p‐values of the association between chronological age and the predicted age obtained after applying the transcriptomic clock to AgeAnno's pseudo‐bulk samples, for each cell type. The p‐values were calculated using an F‐test and adjusted using Benjamini‐Hochberg's method.

Cell type	p‐value	Adj. p‐value (BH)
Astrocytes	1.06·10^−1^	1.06·10^−1^
Endothelial	2.1·10^−2^	3.15·10^−2^
Excitatory neurons	7.09·10^−7^	4.26·10^−6^
Inhibitory neurons	4.35·10^−4^	1.3·10^−3^
Oligodendrocytes	1.47·10^−3^	2.95·10^−3^
OPCs	3.9·10^−2^	4.67·10^−2^

These results demonstrate that, although our clock was trained on bulk RNA‐seq brain samples, it can effectively identify aging‐related differences at the individual cell type level, with particularly strong performance in neurons and oligodendrocytes.

### 2.3 Evaluating Chemical and Genetic in Vitro Perturbations for Rejuvenating Effects

**Figure 4:**
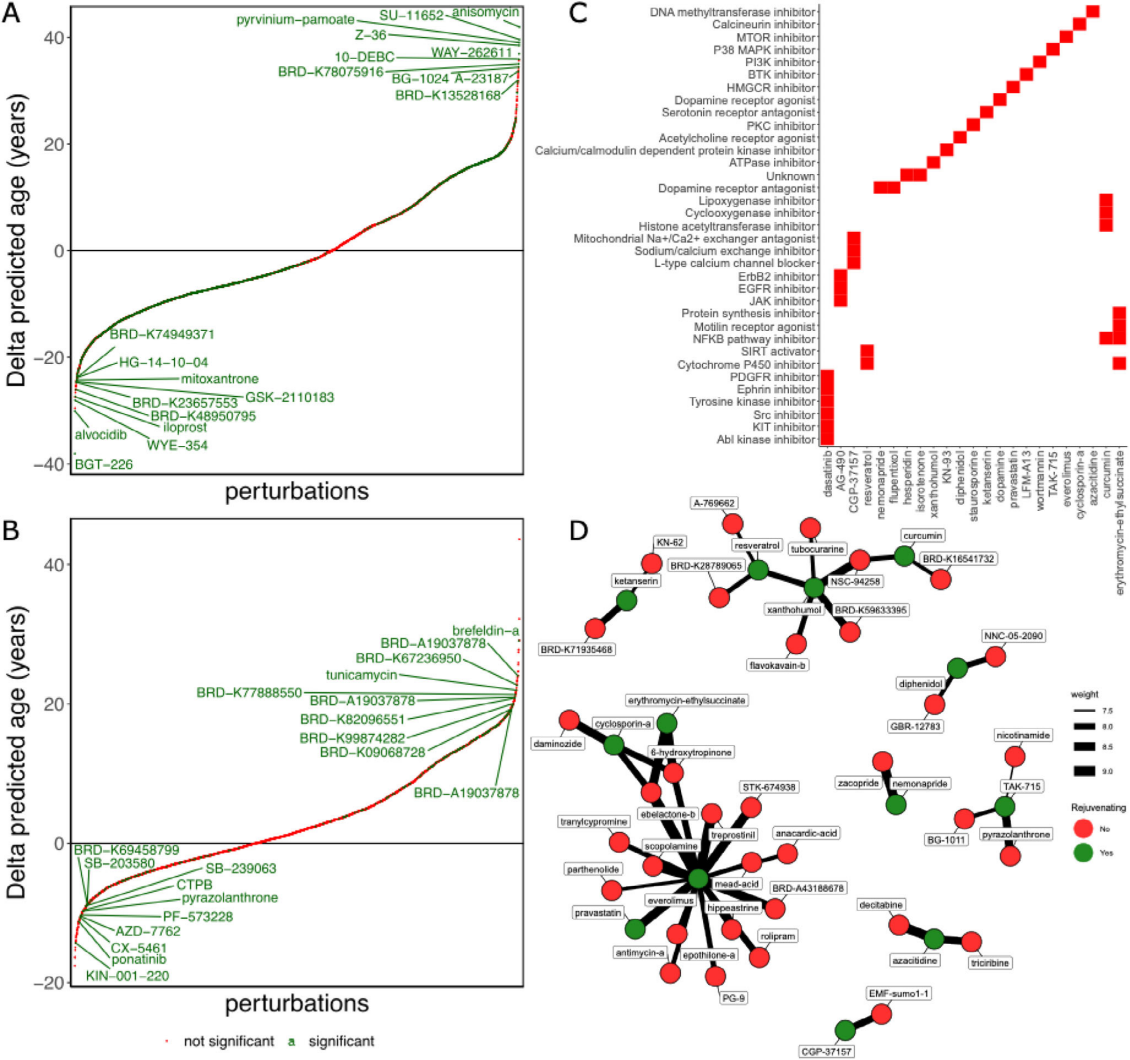
Interventions that shift the transcriptomic age. A and B. Delta age produced by each perturbation (median(treated) minus median(untreated)). The perturbations that are statistically significant are shown in green, the not significant ones in red. A) Neural progenitor cells (NPCs). B) Neurons. C and D. Predicted compounds with known rejuvenation effects. C) Mechanism of action of all predicted compounds that have been shown to extend lifespan in animal models. D) Structural similarity of predicted compounds that are known to extend lifespan (red) to those without experimental validation (green). The line size corresponds to ten times the Tanimoto similarity between the SMILES representations of the connected compounds. Relationships are only shown if the Tanimoto similarity is greater or equal to 0.5 (weight greater or equal 5).

After demonstrating that our clock can identify age‐related differences at the cell type level, we applied it to a combination of Library of Integrated Network‐Based Cellular Signatures (LINCS) L1000 chemical perturbation data^[^ ^46^ ^]^ and several genetic perturbation datasets (Table S6, Supporting Information) for different brain cell types, specifically neurons and NPCs. In particular, we selected 4047 and 5770 genetic and chemical perturbations in neurons and NPCs, respectively. Using this data, we sought to determine whether our clock can identify rejuvenating interventions based on significant transcriptional age differences before and after perturbation (**Figure** **4**). A two‐sided t‐test comparing the predicted ages before and after each perturbation was conducted, identifying 971 unique perturbations that significantly rejuvenated the transcriptomic profiles of NPCs, and 68 in neurons (Figure 4; Tables S7 and S8, Supporting Information respectively, FDR ≤ 0.05, log_2_FC < 0). These perturbations were produced by 411 unique perturbagens in NPCs, and 67 perturbagens in neurons (Tables S9 and S10, Supporting Information).

In NPCs, the top 5 perturbations that induced more transcriptomic rejuvenation — measured as delta age (treated minus untreated) — were BGT‐226, alvocidib, WYE‐354, iloprost, and BRD‐K48950795 (Figure 4A, **Table** **3**). BGT‐226 is a pan‐PI3K and mTOR inhibitor that underwent clinical trials for cancer but was never approved for clinical use.^[^ ^47^ ^]^ Although its effects on aging have not been assessed to our knowledge, several anti‐aging compounds such as rapamycin, everolimus or wortmannin share a similar mechanism of action.^[^ ^48^ ^]^ Alvocivid, a cyclin‐dependent kinase inhibitor that is FDA‐approved for acute myeloid leukemia, has shown behavioral and cognitive improvements in AD mouse models.^[^ ^49^ ^]^ WYE‐354, like BGT‐226, is an mTOR inhibitor with demonstrated anticancer efficacy in pre‐clinical studies, but has not progressed to clinical approval.^[^ ^50^ ^]^ Iloprost, a prostacyclin analog approved for idiopathic pulmonary arterial hypertension, has not been linked to aging‐related benefits.^[^ ^51^ ^]^ Lastly, BRD‐K48950795 is an experimental compound with no known therapeutic application.

**Table 3:** Top 15 drugs for neural progenitor cells (NPCs) and neurons in terms of average Δ age across the perturbations that used that drug (different doses and treatment times).

NPCs		Neurons	
Drug	Mean Δ age (years)	Drug	Mean Δ age (years)
BGT‐226	−19,49	KIN‐001‐220	−14,32
alvocidib	−30,06	ponatinib	−11,24
WYE‐354	−28,06	CX‐5461	−6,84
iloprost	−27,32	AZD‐7762	−10,44
BRD‐K48950795	−26,05	PF‐573228	−10,30
BRD‐K23657553	−24,69	pyrazolanthrone	−9,76
GSK‐2110183	−24,59	CTPB	−9,69
mitoxantrone	−24,25	SB‐239063	−9,05
HG‐14‐10‐04	−16,04	SB‐203580	−9,03
BRD‐K74949371	−24,19	BRD‐K69458799	−8,84
PHA‐767491	−13,63	XMD‐892	−8,69
ST‐4029573	−23,41	JNJ‐38877605	−8,38
BRD‐K76236182	−23,26	BRD‐K83620635	−8,16
NVP‐TAE684	−22,86	zacopride	−7,64
AZD‐6482	−22,80	BRD‐A35825362	−7,30

In neurons, top 5 rejuvenating perturbations were KIN‐001‐220, ponatinib, CX‐5461, AZD‐7762, and PF‐573228. These five compounds, though not being linked to aging, have shown efficacy against several types of cancer.^[^ ^52^, ^53^, ^54^, ^55^, ^56^ ^]^

We focused on chemical perturbations to assess if any of them had demonstrated rejuvenating properties. For this, we queried the DrugAge database^[^ ^57^ ^]^ with the predicted perturbations. As a result, three compounds predicted in neurons (dopamine, nemonapride, and TAK‐175) and 20 compounds predicted in NPCs were found to significantly extend lifespan in animal models, according to DrugAge (Figure 4C). Interestingly, a larger number of predicted rejuvenating compounds emerged in NPCs. This may reflect the higher plasticity of progenitor cells or the increased responsiveness of stem‐like cells to pharmacological modulation, which is consistent with the broader research focus on reversing stem cell decline as a strategy to enhance tissue regeneration and longevity.^[^ ^58^ ^]^ The identified perturbations have diverse mechanisms of action, many of which are directly related to the hallmarks of aging (Figure 4C).^[^ ^59^ ^]^ Curcumin and erythromycin‐ethylsuccinate, for instance, are modulators of inflammation, thus targeting directly the detrimental effect of age‐related neuroinflammation on brain function.^[^ ^60^ ^]^ In addition, brain aging is associated with hypermethylation of CpG islands and the progressive loss of histone acetylation.^[^ ^61^, ^62^ ^]^ Azacitidine can counteract these dysregulations by inhibiting DNA methyltransferases. Our predictions with known rejuvenating effects also contain a modulator of ion homeostasis, KN‐93, which is frequently dysregulated due to altered levels of ion channels.^[^ ^63^ ^]^ Finally, the mTOR inhibitor everolimus has been predicted.^[^ ^48^ ^]^

Although several compounds predicted by our model have been shown to extend lifespan, the vast majority of perturbations has not been studied in the context of health‐ or lifespan extension. Moreover, many predicted compounds are still experimental, which is evidenced by the generic names assigned to them (e.g., compound names starting with ‘BRD‐‘), and their mechanism of action remains elusive. To shed light on their potential modes of action, we sought to interrogate the relationship between these compounds and predicted perturbations with known rejuvenating effects. In lieu of functional information, we compared their 2D structures grounded on the fact that similar compounds may possess similar functions.^[^ ^64^ ^]^ To achieve that, we computed the Tanimoto similarity (TS), where the size of the compound intersection is divided by the size of the smaller descriptor, between the SMILES representation of any two predicted compounds and retained those showing at least intermediate similarity (TS greater or equal 0.75). As a result, we identified several structurally similar compounds in NPCs while no significant association could be found in neurons (Figure 4D). In total, 34 predicted compounds could be structurally linked to 13 perturbations with known rejuvenating effects. The largest cluster of compounds is centered around the mTOR inhibitor everolimus. Other compounds belonging to this cluster are pravastatin, cyclosporin‐a, and erythromycin‐ethylsuccinate, which also appear in DrugAge as lifespan extending, and tranylcypromine, a compound that has been shown to have neuroprotective effects against amyloid‐β‐induced toxicity.^[^ ^65^, ^66^ ^]^ The second largest cluster was centered around resveratrol, xanthohumol, and curcumin, the three of them nutraceuticals that appear in DrugAge as lifespan extending and that possess antioxidant, anti‐inflammatory, and anti‐cancer properties.^[^ ^67^, ^68^, ^69^ ^]^ In summary, the structural similarities identified between compounds with and without a demonstrated ability to extend lifespan support the hypothesis that all of these compounds indeed display rejuvenating effects.

### 2.4 Transcriptional Age is Related to Neurological Disorders

Although our clock accurately predicted the chronological age of brain sample donors based on the expression of the 365 genes and successfully identified chemical perturbations that are able to expand lifespan in animal models, it has not yet been shown that these perturbations could also counteract neurodegeneration. Despite the fact that lifespan is the main readout in most current rejuvenation studies, the extension of healthspan requires restoring the functional capacity of tissues. To investigate this, we first used our clock to predict the age in samples of donors suffering from neurodegenerative disorders compiled from the Accelerating Medicine Partnership: Alzheimer's Disease (AMD‐AD) database,^[^ ^38^ ^]^ as well as from the Traumatic Brain Injury (TBI)^[^ ^40^ ^]^ and Genotype‐Tissue Expression (GTEx) databases.^[^ ^39^ ^]^

**Figure 5:**
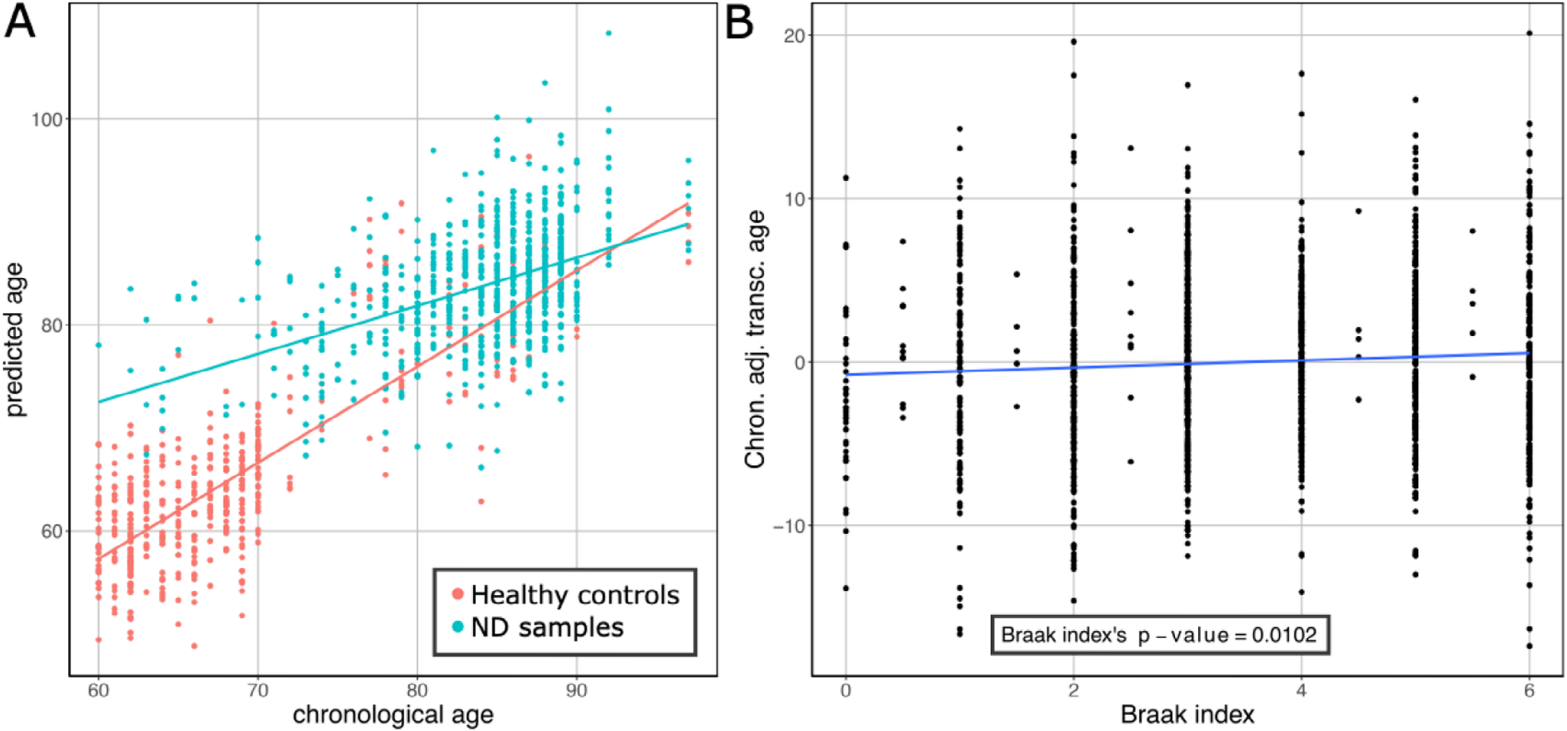
Relationship between predicted age (transcriptional age) and brain functionality. A) Differences in predicted age in healthy controls of the test set (pale red) and samples originating from individuals suffering from neurodegeneration (Braak index ≥ 4). B) Relationship between the Braak index and the adjusted transcriptional (predicted) age. The transcriptional age was adjusted by regressing out the chronological age, to account for potential confounding effects. The p‐value displayed corresponds to the F‐test for Braak index derived from a linear model using adjusted predicted age as response variable and Braak index as covariate.

To start with, we observed that neurodegenerative samples exhibited a transcriptional age higher than that of control samples (**Figure** **5**A). This was particularly evident in samples coming from donors aged 60 to 70, with the neurodegenerative samples having a transcriptional age 15.23 years higher than the healthy individuals at 60 years old. As chronological age increased, the transcriptional ages of neurodegenerative and control samples converged. An ANCOVA test validated the significance of these differences, indicating both neurodegeneration status and the interaction between neurodegeneration status and chronological age to be significant predictors of transcriptional age (p‐values of 2.18·10^−48^ and 1.92·10^−23^).

Next, we evaluated whether transcriptional age is associated with neurodegeneration stage. For this, we used the Braak index, a metric that classifies pathology severity in Parkinson's and Alzheimer's diseases. A linear model was fitted using predicted age as the response variable, Braak index as the main predictor, and chronological age as a covariate to adjust for potential confounding (Figure 5B). The Braak index to was statistically associated with transcriptional age (p‐value = 0.0102).

To explore age‐dependent effects, we repeated the analysis across chronological decades from 60 to 100 years — the range covering all neurodegeneration samples (Figure S7, Supporting Information). From ages 60 to 70, the association remained significant (p‐value = 1.32·10^−3^, coefficient = 1.082). The relationship weakened with increasing age, between 70 and 80, the association was marginally non‐significant (p‐value = 7.03·10^−2^, coefficient = 0.346), From 80 to 90 it was not significant (p‐value = 0.284, coefficient = 0.12), and between 90 and 100, no association was observed (p‐value = 0.965, coefficient = −0.016).

Overall, these results demonstrate that individuals presenting brain functional decline exhibit a transcriptomic profile similar to that of individuals with higher chronological age. In addition to our clock's ability to predict accurately the age in healthy individuals, it can also detect the transcriptomic differences characterizing neurodegeneration. Moreover, our clock revealed that the stages of neurodegeneration significantly contribute to an increased predicted age, indicating that the degree of brain functional decline significantly correlates with a higher transcriptional age. This increase in transcriptional age among individuals suffering from neurodegeneration is more marked in younger subjects, suggesting that neurodegeneration accelerates the aging process.^[^ ^35^ ^]^ These results support that differences in brain transcriptional age imply differences in brain function, which further implies that perturbations predicted by our clock to rejuvenate the transcriptome may have the potential to improve brain function.

To support this claim, we set out to investigate whether the predicted compounds in neurons and NPCs have been used for treating neurological disorders. To achieve that, we collected a list of drugs that have been used in clinical trials or as a treatment for 1945 diseases, including 155 neurological disorders such as Alzheimer's Disease, Parkinson's Disease or stroke, from the Therapeutic Target Database (TTD).^[^ ^70^ ^]^ Due to ambiguities of compound names, we associated the predicted compounds to drugs in the TTD based on the TS of their SMILES representations. In order to avoid false associations, we applied a strict cutoff of 0.85, which is generally accepted as high similarity. As a result, the compounds predicted to rejuvenate NPCs were found to have been applied to 8 neurological disorder categories, Alzheimer's disease, amyotrophic lateral sclerosis, fragile X syndrome, neurological disorder, neurofibromatosis type 1, neurogenic bladder dysfunction, postherpetic neuralgia, and schizophrenia. In neurons, the only potentially rejuvenating compound that has been used in neurological disorders was dopamine, for Parkinson's disease.

Altogether, these results support the relationship between transcriptional age and brain functioning.

### 2.5 Drugs Predicted by Our Clock to Rejuvenate the Transcriptome Reduced Anxiety in Older Mice and Showed a Trend Toward Improved Memory Performance

Among the brain‐rejuvenating interventions that were individually predicted by our clock, an interesting subset includes 5‐azacytidine, tranylcypromine, and JNK‐IN‐5A (TCS JNK 5a), which influence epigenetic regulation.^[^ ^71^ ^]^ As mentioned earlier, 5‐azacitidine was one of the drugs we predicted as rejuvenating, which appeared as lifespan‐extending in DrugAge, while tranylcypromine has considerable structural similarity to everolimus (Figure 4C,D). On the other hand, JNK‐IN‐5A is a selective inhibitor of JNK2 and JNK3, with IC_50_ values of 316.23 and 199.52 nm, respectively.^[^ ^72^ ^]^ This inhibitory potency is lower compared to covalent inhibitors like JNK‐IN‐8, which exhibits IC_50_ values of 4.7, 18.7, and 0.98 nm for JNK1, JNK2, and JNK3, respectively.^[^ ^73^ ^]^ Although JNK‐IN‐8 was not included in the dataset used to predict rejuvenating interventions, its shared targets and greater efficacy made it a compelling candidate for experimental validation. Consequently, we evaluated the combination of 5‐azacytidine, tranylcypromine, and JNK‐IN‐8 in aged mice to assess both molecular and functional brain rejuvenation.

**Figure 6:**
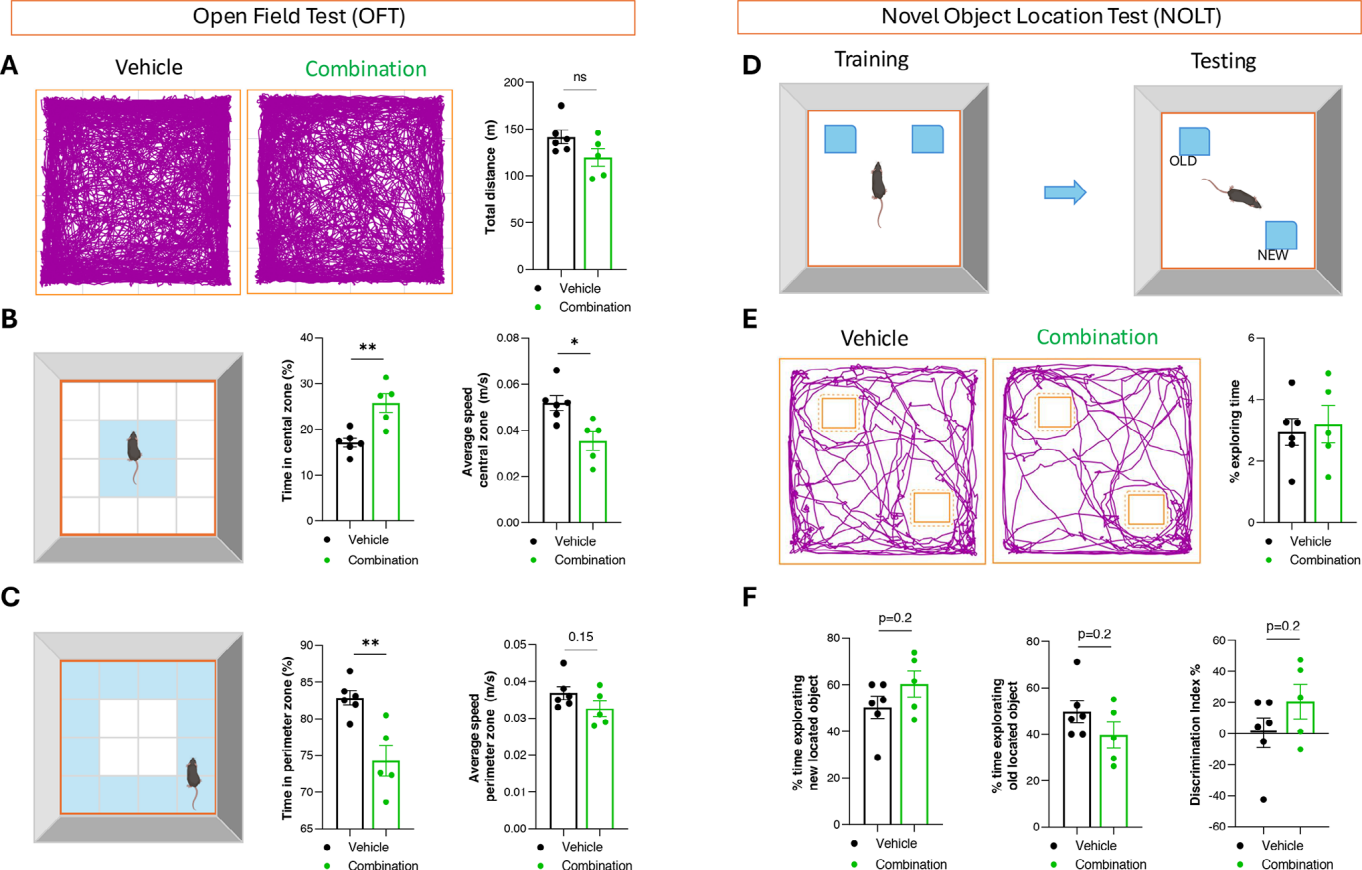
Old mice treated with the combination of three compounds show reduced anxiety and improved spatial memory. A) Track plot of the Open Field Test (OFT) showing the total distance (in meters) travelled by animals treated with either vehicle or the three‐compound combination. B,C) Representative images of the Open Field square, highlighting the two areas to be analyzed in Any‐maze, the center zone and the perimeter (colored blue). Graphs show the percentage of time spent in each area and the average speed (in meters per second) in both zones for the two groups of animals. D) Diagram of the Novel Object Location Test (NOLT), indicating the precise location of objects during the training period and the new position for one object during the testing period. E) Track plot of the NOLT showing the layout of the animals. The percentage of exploring time was calculated by dividing the time the animals spent exploring the object in the new position by the total duration of the test. F) Percentage of time spent exploring the object in the new and old locations for both vehicle‐treated and treated animals. n = 6 vehicle‐injected animals (DMSO) and n = 5 animals injected with the three‐compound combination (5‐azacytidine, tranylcypromine, and JNK‐IN‐8), administered intraperitoneally 8 times. ^*^ *p* < 0.05, ^**^ *p* < 0.01, Student's t‐test.

The combination of these three compounds or vehicle was administered intraperitoneally for 4 weeks to aged mice (18 months) to evaluate behavioral and cognitive functions. Mice treated with the combination of the three compounds showed differences in their exploratory behavior in an Open Field Test (OFT) compared with animals injected with the vehicle. While the total distance traveled remained unchanged between the two groups (**Figure** **6**A), time spent in the central zone was significantly higher in treated animals, and their average speed in the central zone was lower (Figure 6B). Furthermore, time spent in the periphery was shorter in the treated animals, as was their average speed (Figure 6C). An increase in central locomotion or time spent in the central part of the device, without a change in total locomotion, is interpreted as an anxiolytic‐like effect.^[^ ^74^ ^]^ Together, these results suggest that this combination reduces anxiety levels compared to vehicle‐treated mice.

The Novel Object Recognition Test (NORT) was also conducted to evaluate both short‐ and long‐term memory in the animals. Vehicle‐ and treated animals were compared based on their ability to recognize when a familiar object had been replaced by a new one, showing similar performance between the two groups (Figure S8, Supporting Information). Next, the Novel Object Location Test (NOLT) was conducted to assess spatial long‐term memory, as healthy animals typically explore displaced objects more than those in their familiar locations (Figure 6D).^[^ ^75^ ^]^ Although exploratory behaviour did not differ between groups (Figure 6E), animals administered the three‐compound combination tended to spend more time exploring the newly relocated objects compared with vehicle‐treated animals (Figure 6F). Consistent with this, the time spent exploring the old, familiar object was lower in the treated animals (Figure 6F). Finally, the discrimination index, calculated as the ratio of time exploring new object/time exploring old object), tended to be higher in animals administered the three‐compound combination compared with vehicle‐treated mice (Figure 6F). Therefore, these results indicate that animals treated with the combination of the three compounds have slightly increased spatial memory, though the difference was not significant.

### 2.6 Transcriptomic Profiles of Mice Treated with 5‐Azacytidine, Tranylcypromine and JNK‐IN‐8 Show Brain Rejuvenation

**Figure 7:**
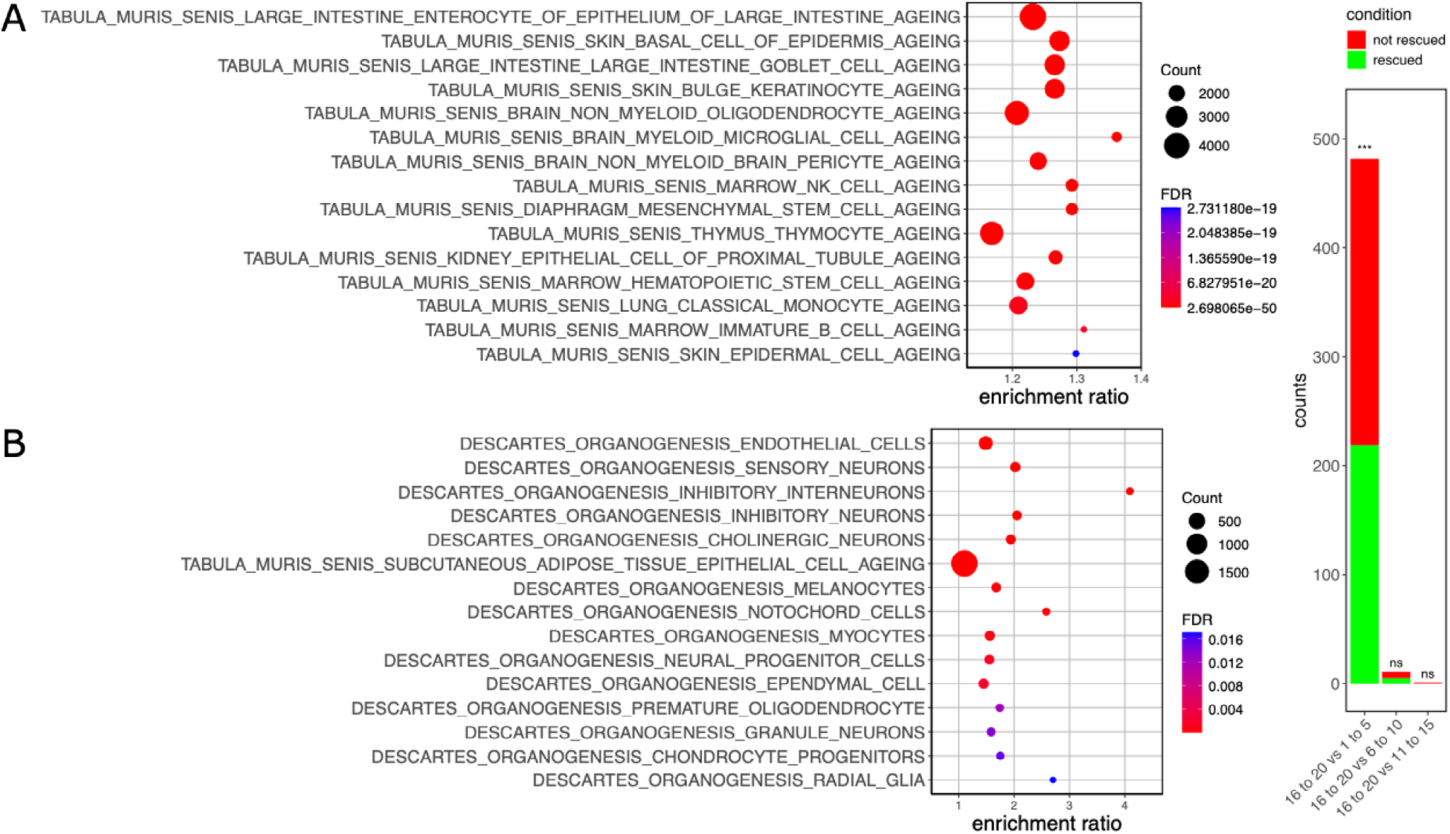
Transcriptomic rejuvenation of old mice after treatment with 5‐azacytidine, tranylcypromine, and JNK‐IN‐8. A,B. Top 15 enriched gene sets in the differentially expressed genes identified in old mice after treatment with 5‐azacytidine, tranylcypromine and JNK‐IN‐8, determined by NEAT enrichment, and sorted according to FDR. The gene sets used as a reference were obtained from Tabula Muris Senis. In the X axis it is represented the ratio between the number of links between either the differentially expressed genes the gene sets used as reference in our enrichment, and the expected number of links in the absence of enrichment. A) Enrichment performed with the downregulated differentially expressed genes or B) Enrichment performed with the upregulated differentially expressed genes. C) The differentially expressed genes of mice aged 16 to 20 months versus either mice aged 1 to 5, 6 to 10, or 11 to 15 months were determined, respectively (FDR ≤ 0.05). Then we assessed how many genes were rescued (the log2 fold changed moved toward the younger phenotype) after a treatment with 5‐azacytidine, tranylcypromine, and JNK‐IN‐8. The significance of the enrichment in rescued genes was assessed by a right‐tailed Fisher exact test (FDR ≤ 0.05, ^***^ *p* ≤ 0.001, ns > 0.05).

The cortical transcriptome of mice treated with either the combination of 5‐azacytidine, tranylcypromine, and JNK‐IN‐8 or the vehicle was analyzed through RNA sequencing, identifying 493 differentially expressed genes (adjusted p‐value ≤ 0.05, Figure S9, Supporting Information). These genes were categorized into overexpressed and repressed subsets based on their log2 fold change, followed by NEAT enrichment analysis for each subset, using as reference MSigDB gene sets.^[^ ^76^ ^]^ In the downregulated subset, 161 enriched gene sets were identified (**Figure** **7**A; Table S11, Supporting Information). Processes linked to brain aging were significantly overrepresented within this subset, as confirmed by a Fisher exact test (p‐value = 0.03). In contrast, analysis of the upregulated subset revealed 21 significantly enriched gene sets (Figure 7B; Table S12, Supporting Information). While none were directly associated with brain aging, several were related to neural cell types.

Next, we aimed to determine whether the treatment administered to our mice could shift their brain transcriptome toward a younger phenotype. To investigate this, we used Tabula Muris Senis brain bulk expression dataset to identify differentially expressed genes across age groups.^[^ ^77^ ^]^ Specifically, we compared samples from mice aged between 16 and 20 months, which include the age range of our study subjects, with samples from three younger age brackets, 1–5, 6–10, and 11–15 months, respectively. For each comparison, we assessed the extent to which our treatment restored the expression of age‐associated genes, where “rescue” was defined as a post‐treatment log_2_ fold‐change that moved expression closer to that of the younger group. We evaluated the enrichment of rescued genes for statistical significance and found a significant enrichment for genes differentially expressed between the 16–20 and 1–5 month age groups (Figure 7C). This suggests that a notable proportion of age‐related gene expression changes were shifted toward a younger phenotype by the treatment.

Altogether, these results show that a selection of compounds predicted by our clock to transcriptionally rejuvenate the brain indeed produce rejuvenation at the transcriptome level in aged mice, which translates into an improvement of the age‐derived functional decline.

## 3 Discussion

In this study we presented a computational platform designed to identify interventions that can transcriptionally rejuvenate different human brain cell types, which we implemented as an R package, brainAgeShiftR. At the core of this platform is a brain‐specific transcriptional aging clock that showed high accuracy and generalizability in predicting the chronological age of healthy controls, based on the expression of 365 genes. Though for our purpose, we aim for a clock that is able to detect changes in age rather than to predict it accurately, the metrics it achieved are comparable to other high‐quality brain transcriptomic clocks. For instance, the neural transcriptomic clock developed by Martinez‐Magaña et al. shows a Pearson correlation of 0.89 between transcriptional age and chronological age and a RMSE or 5.55 when tested in prefrontal cortex transcriptomic profiles.^[^ ^78^ ^]^ On the other hand, RNAAgeCalc shows a Pearson correlation of 0.68 and a RMSE or 9.09 the same conditions.^[^ ^79^ ^]^ Ours shows a Pearson correlation of 0.902 and a RMSE of 6.103 in the internal test set, while in the external validation set the Pearson correlation was 0.93 and the RMSE 7.171.

By comparing the predicted ages of the healthy controls to those of individuals suffering with neurological conditions, we observed that functional impairments are reflected in the transcriptional age of brain cells. Furthermore, the degree of neurodegeneration, as measured by the Braak stage, was significantly associated with increased transcriptional age. These findings show that transcriptional age is negatively correlated with brain function, supporting the view of neurodegeneration as a form of accelerated aging.^[^ ^10^, ^12^ ^]^ Therefore, our model captures aspects of functional brain fitness, opening the possibility of utilizing it to identify potential treatments for neurodegenerative diseases and other conditions affecting brain function. While recent approaches have proposed training transcriptomic clocks on both healthy and pathological samples to reduce bias and improve calibration across disease states,^[^ ^80^ ^]^ our aim is to detect perturbation‐induced deviations from a normative aging trajectory. In this context, training exclusively on healthy individuals provides a clear reference for identifying acceleration or rejuvenation of the aging process.

The 365 genes used by our model represent the expression signatures of brain aging. While 91 of these genes were directly involved in brain‐related processes, a NEAT enrichment^[^ ^43^ ^]^ revealed that none of these processes were significantly enriched. Instead, these genes showed significant enrichment in biological processes related with mitosis, DNA repair, DNA replication, chromatin dynamics, and RNA transcription and processing. Indeed, genomic instability is a hallmark of both healthy and pathological aging, not only in the brain but in other tissues.^[^ ^59^, ^81^ ^]^ Despite a considerable number of brain‐related genes in our model, it is likely that their diverse functional categorization makes it difficult to achieve statistical significance in specific GO terms.

Our model was built based on human brain bulk transcriptomics due its wider availability compared to human single cell RNA‐seq, which, while ideal for capturing brain's cellular heterogeneity, is still an emerging approach and represents the direction the field is moving toward.^[^ ^82^ ^]^ However, bulk RNA‐seq provides an averaged representation of all brain cell types, meaning that the age‐related processes identified are those shared across cell types. When we applied our bulk transcriptome‐trained model to single‐cell brain pseudo‐bulk data, we found that the trend of increased predicted age in older individuals was consistent across all cell types. This trend was statistically significant in excitatory neurons, inhibitory neurons, and oligodendrocytes. In astrocytes, endothelial cells, and OPCs, while the median predicted age increased in older samples, the high variability within the group made it difficult to find achieve statistical significance. Additionally, the scarcity of endothelial cells in the brain meant they were detected in fewer samples, reducing the statistical power and hampering the detection of significance. However, the relationship between chronological and transcriptional ages was found to be significant for all the cell types. Despite the challenges associated with scRNA‐seq, such as low coverage and dropout events,^[^ ^83^ ^]^ our model successfully captured aging trends in individual cell types. Though the prediction of ages was not as accurate as with bulk data, being able to detect the aging trends in single cell types renders our model apt for the identification of interventions that modulate transcriptional age in cultured brain cell types.

As mentioned earlier, our model reveals a significant positive association between neurodegeneration stage and predicted age, linking increased transcriptional age to decreased functional performance. Interestingly, these differences are pronounced at earlier ages, progressively diminishing as aging advances. This observation invites parallels with the fact that neurodegeneration‐like lesions can be found in cognitively unimpaired older individuals.^[^ ^84^, ^85^ ^]^ Under this hypothesis, neurodegeneration would be a form of accelerated aging, where the affected subjects reach a physiological state that healthy individuals typically attain at a higher chronological age. This has indeed been observed with several epigenetic clocks.^[^ ^34^, ^35^, ^86^ ^]^ According to our model, the largest gap in transcriptional age between individuals with neurodegeneration and healthy individuals occurs between ages 60 and 70. It is likely that these individuals experienced an acceleration during their preclinical phase, transitioning from healthy aging to pathological aging, as it has been reported in mice.^[^ ^87^ ^]^ Shifting back to a younger transcriptomic profile might help to improve the phenotype of neurodegenerative diseases.^[^ ^88^ ^]^

Based on this principle, and on the ability of our model to capture aging differences in individual cell types, with particular efficacy in neurons, we used LINCS L1000 data to identify chemical perturbations that could rejuvenate either neural progenitor cells or neurons. In fact, several of these predicted chemical perturbations in both cell types have been previously shown to extend the lifespan of animal models, although their mechanisms of action are vastly different. This observation is in accordance with previous studies demonstrating that brain aging is a highly heterogeneous process^.[^ ^89^, ^90^, ^91^ ^]^ The rejuvenating chemical compounds identified by our platform were significantly enriched in drugs used for the treatment of neurological disorders, supporting that by shifting brain transcriptome toward a younger state, its function might improve. Interestingly, well‐known rejuvenating compounds like rapamycin (sirolimus) and metformin were not predicted as rejuvenating by our clock. Rapamycin, indeed, produced a significantly higher transcriptional age in NPCs. A recent literature screening showed that rapamycin treatment did not produce significant improvements in neurological systems.^[^ ^48^ ^]^ Rapamycin was shown to suppress differentiation of neural stem cells, which could explain the higher transcriptional age in NPCs.^[^ ^92^, ^93^ ^]^ On the other hand, other mTOR inhibitors such as everolimus were detected as rejuvenating. Metformin, however, produced rejuvenation in neurons, but this shift was not statistically significant. It is likely that our model, due to the linearity assumption, is not capable of capturing the whole extent of the alterations that happen along aging, focusing only on the ones that are linearly related to the chronological age.

As a proof of concept of our approach, we tested three predicted compounds — 5‐azacytidine, tranylcypromine, and JNK‐IN‐8 — in aged mice. The treatment significantly reduced anxiety, and improved memory in aged mice, addressing well‐known aging‐associated alterations.^[^ ^94^, ^95^ ^]^ At the molecular level, it significantly restored the younger phenotype. Notably, all three compounds are epigenetic regulators—a category to which several compounds identified by our platform belong—highlighting the promising role of such drugs in counteracting aging.^[^ ^96^ ^]^ Interestingly, this combination of compounds has previously been used to generate human chemically induced pluripotent stem cells (hCiPS) through staged reprogramming.^[^ ^71^ ^]^ In particular, these compounds were used in stage II of this protocol, shifting the epigenetic landscape to a more plastic state that permits dedifferentiation in later stages.^[^ ^71^ ^]^ This plasticity resembles tissue regeneration programs found in organisms like the axolotl.^[^ ^97^, ^98^ ^]^ While partial cell reprogramming is a recognized rejuvenation strategy,^[^ ^99^ ^]^ its therapeutic potential is limited by the risk of tumorigenesis.^[^ ^28^ ^]^ Although our validation demonstrates the platform's potential to identify brain‐rejuvenating compounds, it was limited to this combination of three molecules in mice. The hundreds of compounds predicted by our platform require validation across diverse multiple biological systems to assess their efficacy, offering extensive opportunities for future research and therapeutic development. Another limitation is that the majority of the data used for our in silico screening comes from LINCS L1000 chemical perturbation profiles, which include only neurons and NPCs, the latter representing a relatively small fraction of cells in the human brain. However, NPCs play a critical regenerative role, and their functional decline significantly contributes to brain aging. This key position makes NPCs an important target for anti‐neurodegenerative therapies^[^ ^100^ ^]^

Our platform's ability to predict epigenetic drugs with rejuvenating properties, including those promoting of a regeneration‐like program, highlights their potential as safer alternatives. Altogether, these results show that by rejuvenating the brain transcriptome it is possible to improve brain function and to protect against neurodegenerative disorders. Consequently, our computational platform represents a valuable resource for identifying interventions that may counteract age‐related brain decline in brain function. The compounds identified by our platform can serve as a foundation for further research to validate both their efficacy and safety.

## 4 Experimental Section

### Description of the Human Postmortem Bulk Brain Expression Data Used

The human postmortem bulk brain expression data used in this study were obtained from GTEx, AMP‐AD, TBI, BrainSeq Phase 1, and BrainSeq Phase 2 projects.^[^ ^38^, ^39^, ^40^, ^41^, ^42^ ^]^

For GTEx, count data were retrieved from brain regions including amygdala, anterior cingulate cortex, caudate (basal ganglia), cerebral hemisphere, cortex, frontal cortex, hippocampus, hypothalamus, nucleus accumbens (basal ganglia), putamen (basal ganglia), spinal cord (cervical) and substantia nigra — excluding the cerebellum due to its distinct transcriptomic profile. Data were downloaded from the GTEx portal on 05/13/24.

From synapse.org, RNA‐seq count data were used from the RNAseq Harmonization Study, which includes samples from the ROSMAP,^[^ ^101^ ^]^ MayoRNAseq^[^ ^102^ ^]^ and Mount Sinai Brain Bank (MSBB)^[^ ^103^ ^]^ cohorts, as well as from the Living Brain Project (LBP). TBI data consisted in gene counts as well. Only those samples with no reported traumatic brain injury (i.e., metadata column “age_at_first_tbi” = 0) were retained. As with GTEx, samples originating from cerebellum were excluded all datasets due to their transcriptomic dissimilarity from other brain regions, as observed in PCAs.

For BrainSeq Phase 1 and BrainSeq Phase 2, only individuals labeled as “control” were included, since the remaining samples came from individuals with schizophrenia. Although BrainSeq Phase 1 and BrainSeq Phase 2 were distinct studies, some individuals were represented in both. To avoid data leakage, any Phase 1 samples originating from individuals also present in Phase 2, were removed as Phase 1 was used as an external validation set.

For all the datasets, samples associated with psychiatric diseases or drug abuse were excluded. Additionally, only samples with an RNA integrity number (RIN) ≥ 6 were retained for analysis.

### Criteria for Defining Healthy Controls

Control definitions varied by dataset based on the available metadata. For GTEx, samples were considered controls if negative for dementia, Parkinson's disease, Alzheimer's disease, and ALS. In the Mayo cohort, control samples were those labeled as such in the clinical diagnosis, and had a Braak stage ≤ 3 and a Thal phase of 0. For MSBB, samples were considered healthy if they had a Braak stage ≤ 3 and a CERAD score indicating no Alzheimer's pathology. In the ROSMAP dataset, control status required both the clinical diagnosis of cognitive status and the final consensus cognitive diagnosis to indicate no cognitive impairment, along with a Braak stage ≤ 3 and a CERAD score showing no Alzheimer's disease. Samples from the Living Brain Project (LBP) were classified as controls if annotated as such. For the TBI dataset, samples were considered controls if they were diagnosed with “No Dementia” according to both the DSM‐IV and NINCDS‐ARDA criteria, with a Braak stage ≤ 3, and a CERAD score showing no Alzheimer's disease. Finally, in BrainSeq Phase 1 and Phase 2, all retained samples were labeled as controls.

The final dataset included samples classified either as controls — which were used for model training and model validation — or cases presenting with evidence of neurodegeneration. Within the neurodegeneration group, only samples with available Braak stage were retained. After filtering, the dataset comprised 2478 control samples from 800 unique individuals, and 1885 neurodegeneration samples from 1045 unique individuals.

### Description of the Genetic and Chemical Perturbation Data Used

The majority of the perturbation data used in this study came from the LINCS L1000 dataset, specifically the chemical perturbation profiles.^[^ ^46^ ^]^ To focus on brain‐relevant effects, only samples derived from neural progenitor cells (NPCs) and neurons were retained. In addition, a curated compendium of both genetic and chemical perturbation datasets conducted in brain cell was incorporated types, which are detailed in Table S6 (Supporting Information).

### Description and Preprocessing of Brain AgeAnno Single Cell RNA‐seq Data

The single‐cell RNA‐seq data from human brain tissue used in this study were obtained from AgeAnno database.^[^ ^45^ ^]^ It was started from the brain Seurat object provided by the authors of the database, accessed on 07/05/2025. Preprocessing was performed with Seurat 5.0.0 R package.^[^ ^104^ ^]^ Quality control was done at the sample level, retaining cells whose mitochondrial gene content fell within three median absolute deviations (MADs) from the median. The same MAD threshold was applied to filter cells based on the number of detected features and total transcript counts. To further refine cell quantity, a linear model was fitted to log_10_(number of counts) versus log_10_(number of features), and excluded cells that fell below the regression line, using an offset of −0.09. Doublets were identified and removed using the DoubletFinder 2.0.4 R package.^[^ ^105^ ^]^

Samples were then integrated using Seurat's sketch integration workflow (n = 5000), clusters were identified using Louvain's algorithm with Seurat's FindClusters function (resolution = 0.2), and were manually annotated using the same marker genes used in AgeAnno. These were CLDN5, EPAS1 and VTN for endothelial cells, GLUL, SOX9, AQP4, GJA1, NDRG2, GFAP, ALDH1A1, ALDH1L1 and VIM for astrocytes, PTGDS, PDGFRA, PCDH15, OLIG1 and OLIG2 for OPCs (oligodendrocytes precursor cells), PLP1, MAG, MOG, MOBP and MBP for oligodendrocytes, SATB2, SLC17A6 and SLC17A7 for excitatory neurons and NRGN, GAD1, GAD2 and SLC32A1 for inhibitory neurons.

For each sample, pseudo‐bulk expression profiles per cell type were computed by summing the counts of cells annotated with the same cell identity.

### Integration and Preprocessing of the Bulk and Pseudo‐Bulk Human Postmortem Datasets

The counts of the human postmortem brain expression data, encompassing all the bulk datasets and the pseudo‐bulk scRNA‐seq dataset, were merged, and genes with zero counts in ≥80% of samples were excluded. Additionally, genes not present in the LINCS expression dataset were excluded, resulting in a final set of 13445 genes. The filtered count matrix was then log_2_‐transformed using the formula log_2_(counts + 1).

To avoid data leakage between training and testing sets, the data split was performed at this stage. Samples were divided into training and testing sets in a 2, 1 ratio, stratified by donor to maintain sample independence and ensuring that each 5‐year interval was represented in the training set. BrainSeq Phase 1 samples were excluded from both sets and reserved for final external validation, as well as pseudo‐bulk scRNA‐seq samples.

After splitting, the log_2_‐transformed data were quantile‐normalized, using the distribution derived from the training set as the reference. Batch effects were then addressed using surrogate variable analysis (SVA). The number of surrogate variables was estimated using the num.sv function from the sva R package (version 3.48.0) with the “leek”, and the surrogate variables were compute using the SmartSVA 0.1.3 R package.^[^ ^106^, ^107^ ^]^ SVA was conducted with a null model containing no covariates, and a full model including only chronological age.

### Transcriptomic Clock Training

The brain‐specific transcriptomic clock was trained in two sequential rounds. In the first round, a generalized linear model (GLM) was fitted to the training set samples of the SVA‐corrected merged dataset. This was performed using the h2o 3.46.0.2 R package,^[^ ^108^ ^]^ specifying a Gaussian family with an identity link function and applying Lasso regularization. This initial model selected 558 genes with non‐zero coefficients.

In the second round, the preprocessing pipeline described previously was repeated, but restricted to the 558 genes identified in the first round. A GLM was then re‐trained again on this reduced dataset using the same settings. This second fitting step further reduced the number of predictors to 365.

This 2‐step approach was achieved to reduce the size of the model by 34.59% with no decline in performance, as assessed by R^2^ and mean average error (MAE). This streamlined model facilitates deployment by requiring only the expression of the initial 558 selected genes. A reference distribution derived from these genes in the training set was used for quantile normalization of new samples, eliminating the need for transcriptomic‐wide data.

### Transcriptomic Clock Validation

Model validation was conducted through internal 10‐fold cross‐validation, stratified by donor, on the training set. Additionally, performance was evaluated on the held‐out test set, a randomly sampled subset of the control samples, and on an external validation set (BrainSeq Phase 1). Model generalizability was assessed using R^2^ and MAE metrics across all evaluation sets.

### Functional Characterization of the Brain Aging Expression Signature

To functionally characterize the 365 genes used as predictors in the transcriptomic clock, the Network Enrichment Analysis Test (NEAT) was applied.^[^ ^43^ ^]^ The *Homo sapiens* FunCoup 5.0 network,^[^ ^109^ ^]^ considering only interactions with a confidence score ≥ 0.75 was used. Gene sets for enrichment analysis were drawn either from Gene Ontology (GO), as provided by org.Hs.eg.db 3.17.0 R package,^[^ ^110^ ^]^ or from the Molecular Signatures Database (MSigDB).^[^ ^76^ ^]^ A gene set was considered significantly enriched if the Benjamini‐Hochberg adjusted p‐value (FDR) ≤ 0.05 and the ratio of observed to expected network‐associated genes (NAB/expected NAB) exceded > 1. Here, NAB represents the number of links between clock genes and genes associated with a given gene set. For determining the genes that were associated to brain‐related processes, was looked for GO biological processes for each gene that contained the words with the following lexemes, “synap‐”, “brain‐”, “cereb‐”, “neur‐”, “dendr”, “axon‐”, “cort‐”, “hippo‐”, “thalam‐”, “medul‐”, or “glia‐”.

### Brain Cell Type Analysis

The transcriptomic clock was applied on the preprocessed pseudo‐bulk counts of each cell type of each sample using h2o.predict function from h2o R package. 108 Two groups of samples were obtained, young, which were the samples aged from 18 to 30, both included, and old from 70, included, onward. A two‐sided t‐test was then conducted for each cell type to assess the statistical significance of the differences in predicted ages between young and old samples.

To assess the alignment between gene expression changes and the coefficients in the clock, the difference in median expression between young and old samples was calculated for each cell type. Quantified the proportion of genes whose expression changes matched the direction of their corresponding clock coefficients was then. Statistical significance of this proportion was evaluated with a permutation test (n = 10000).

### Integration and Preprocessing of the Perturbation Data

The perturbation transcriptomic profiles listed in Table S6 (Supporting Information) were combined with LINCS L1000 Level 3 expression data from brain cell types to form a single dataset. Gene filtering was applied to retain only the 558 genes identified during the first round of the transcriptomic clock training. The resulting matrix was then quantile‐normalized, using the distribution derived from the training samples in the SVA‐processed dataset used during the first round of model training as the reference, taking only into account the 558 genes identified during the first round of training.

### Perturbation Analysis

As with the individual cell data, the ages were predicted in the preprocessed perturbation data using h2o.pedict function from the h2o R package (v3.46.0.2).^[^ ^108^ ^]^ As such, the transcriptional age was computed for each one of the 43840 transcriptional profiles were collected, and assessed the statistical significance of the differences in ages between controls and samples subjected to the same perturbation though a two‐sided t‐test. Samples were considered to be subjected to the same chemical perturbation if they shared perturbation, dose, and treatment time. For genetic perturbation, they were considered to be the same perturbation if they shared gene and perturbation mechanism. P‐values were adjusted using Benjamini‐Hochberg method, across all comparison done within the same cell type. The unique perturbagens involved in the significant perturbations were then determined.

Drugs with demonstrated lifespan extending effects were obtained from DrugAge.^[^ ^57^ ^]^ In particular, only significant changes according to DrugAge were considered regardless of the animal model. The resulting drugs were then matched to perturbations predicted to have a significant age reduction through manual curation to avoid mismatches due to different naming conventions.

Finally, similarity of chemical perturbations with predicted rejuvenating effect was assessed using Tanimoto similarity (TS) of the SMILES representations contained in the LINCS L1000 metadata where the size of the intersection is divided by the size of the smaller compound. Perturbations with a TS greater or equal to 0.75 were considered similar. TS was computed using the ChemmineR 3.56.0 R package.^[^ ^111^ ^]^

### Analysis of the Relationship Between Transcriptional Age and Neurodegeneration

To determine the relationship between higher transcriptional ages and functional decline, transcriptional ages were obtained as previously described for preprocessed samples that were not classified as healthy controls and had a Braak ≥ 4. Conducted an ANCOVA was then to assess both the effect of neurodegeneration status and its interaction with chronological age, using the rstatix 0.7.2 R package.^[^ ^112^ ^]^ The following model was used,

(1) $$age_{t}=age_{c}+ND+ND•age_{c}$$

where *age_t_* and *age_c_* are transcriptional and chronological age, respectively, and *ND* represents neurodegeneration status.

The age difference between the neurodegeneration‐positive individuals and healthy controls at age 60 was calculated by adding the product of 60 and the coefficient of the interaction term (−0.46636) to the coefficient of neurodegeneration status (43.21325).

To evaluate the relationship between neurodegeneration stage and transcriptional age, the transcriptional age was computed for all the samples not classified as controls and with available Braak index information. Chronological age was first regressed out from transcriptional age, and a linear model was fitted with Braak index as the explanatory variable and chronological age‐corrected transcriptional age as the response variable. An F‐test was used to determine the significance of the relationship. Similarly, this significance was assessed within each age decade by fitting separate models for the age groups 60–69, 70–79, 80–89, and 90–99.

To identify chemical perturbations previously used to treat neurological disorders, the complete Therapeutic Target Database (TTD)^[^ ^70^ ^]^ was retrieved and manually selected all entries related to neurological disorders. The Tanimoto similarity (TS) between all drugs in the TTD and predicted compounds was then computed using the ChemmineR 3.56.0 R package.^[^ ^111^ ^]^ A compound‐drug relationship was established if the TS was greater or equal to 0.85.

### Animals

Experiments were performed in wild‐type aged animals from the C57BL/6J strain purchased from Charles Rivers Laboratories (France) at 18 months age. Animal experiments were conducted in accordance with the standards approved by the Animal Care Committee at University of Santiago de Compostela, and the experiments were performed in agreement with the institutional guidelines and the European Union standards for the care and use of experimental animals. Animals were housed and maintained in a controlled environment at 22–24 °C and 55% humidity, on 12 h light/dark cycles, and fed with regular rodent chow and water ad libitum.

### Drug Treatment

The three compounds — 5‐azacytidine (Sigma‐Aldrich #A2385), tranylcypromine‐HCl (Merck #616431) and JNK‐IN‐8 (Sigma‐Aldrich #SML1246) — were each dissolved separately in DMSO (Sigma‐Aldrich #D8418) as the sole solvent at room temperature, achieving concentrations of 49, 50, and 20 mg mL^−1^, respectively. After preparing the solutions, mice were administered the compounds intraperitoneally using a 27G syringe at the following doses, 0,5 mg kg^−1^ for 5‐azacytidine, 1,5 mg kg^−1^ for tranylcypromine, and 2 mg kg^−1^ for JNK‐IN‐8. The drug solutions were combined to achieve the required final dosage for each injection. Injections were administered at a volume of 2.5 µL per gram of body weight, twice per week for one month.

### Behavioral Tests

The week before starting the behavioral tests, animals were placed in the experimental room with faint light to adapt them to the light conditions. Animal performance in all the tests was recorded with a camera and the *Free2x Webcam recorder* software.

### Open Field Test (OFT)

The open field test was used to evaluate spontaneous locomotor activity, emotional response, and exploratory behavior. Briefly, animals were placed in the empty field (45 × 45 cm cage) during 60 min and allowed to move freely and recorded. Total distance travelled (m) and speed (m/s), and time (s) spent in the center zone (20 × 20 cm) and perimeter area were measured.

### Object Recognition Tests

Two different object recognition tests were used to evaluate short‐term memory, long‐term memory, and spatial memory.

The first test performed was the Novel Object Recognition Test (NORT) for short and long‐term memory evaluation. Animals were placed in the field with two identical objects and were allowed to explore them for 10 min. After this time, mice were put back in their home cages and one of the objects was removed to put in its place a new one with different shape. 1 h after the first trial, animals were placed again in the field and allowed to explore the objects for 10 min. Performance in this trial was used to analyze short‐term memory. 24 h after the NORT for short‐term memory evaluation, the test was repeated for the study of long‐term memory. Mice were put again in the field with the same original object and a new object different from the previous one and allowed to explore for 10 min.

The second test performed was the Novel Object Location Test (NOLT) for spatial memory evaluation. After the exposure of the animals to two identical objects in the field for 10 min, the location of one of the objects was changed. 24 h later, animals were placed again in the field so they could explore the objects again for 10 min.

Exploratory behavior, represented by the percentage of total time spent by the animals exploring both objects, and preference for the new object, represented by percentage of time spent exploring the new object and the discrimination index, were measured in both tests.

### Behavioral Tests Analysis

All the tests were analyzed using ANY‐maze software. Statistical analysis was performed using GraphPad Prism, and data were expressed as mean ± S.E.M. Statistical differences regarding group were assessed by unpaired t‐test. Statistical significance was set at p < 0.05, and normality and outlier test were performed for each dataset.

### Cortex Bulk RNA Extraction

Following the behavioral tests, mice received an additional two weeks of treatment with the same three‐compound regimen described previously. Afterward, mice were sacrificed, and cortical tissue was collected for RNA extraction. Between 20 and 30 mg of cortex were immediately frozen upon dissection. Tissue was homogenized in 500 µl of TRIzol (Invitrogen) using a TissueLyser II (Quiagen). Next steps of the RNA extraction were performed following the TRIzol manufacturer´s instructions. RNA pellets were resuspended in 50 µl of RNase‐free water and further purified using the GeneJet RNA Cleanup and Concentration Micro kit (Thermo Scientific, K0841). RNA concentration was determined using the Qubit RNA HS Assay Kit (Thermo Fisher Scientific). RNA quality was assessed with Agilent RNA 6000 Nano and Pico Chips (Agilent Technologies), and RNA integrity numbers (RINs) were obtained using the Agilent 2100 Bioanalyzer.

### Cortex Bulk RNA‐seq Library Preparation, Sequencing and Alignment

TruSeq Stranded Total RNA libraries were prepared with the Ribo‐Zero Globin kit and TruSeq RNA CD Index Plate (both from Illumina Inc.) following the manufacturer's instructions (Part #15031048 Rev. E). In particular, starting with 500 ng of total RNA, rRNA and globin mRNA were depleted, and the remaining RNA was purified, fragmented, and primed for cDNA synthesis. First‐strand cDNA synthesis was performed using SuperScript‐II Reverse Transcriptase (Thermo Fisher Scientific, Waltham, MA) under the following conditions, 10 min at 25 °C, 15 min at 42 °C, 15 min at 70 °C, and pause at 4 °C. Second‐strand synthesis was carried out with Illumina reagents at 16 °C for 1 h, followed by A‐tailing and adaptor ligation. Finally, RNA was amplified by PCR (30 s at 98 °C, 15 cycles of 10 s at 98 °C, 30 s at 60 °C, 30 s at 72 °C, 5 min at 72 °C, and pause at 4 °C). The libraries were sequenced in a NovaSeq‐6000 (Illumina Inc.), generating a minimum of 100 million paired‐end 150 nt reads. Reads were subsequently aligned to the GRCm39 genome using STAR v2.7.1^[^ ^113^ ^]^ with GENCODE M36 annotation. Gene‐level expression quantification was conducted using featurecounts from the Subread package (v2.0.8).^[^ ^114^ ^]^

### Tabula Muris Senis data

The brain bulk data was obtained using TabulaMurisSenisData R package (v3.20).

### Differential Expression Analysis

To perform differential expression analysis, only genes were retained that had at least 5 raw counts in at least two samples. Differential expression testing was performed using the DESeq function from the DESeq2 R package (v1.36.0).^[^ ^115^ ^]^ In particular, a Wald‐test was performed, and dispersion was estimated using the glmGamPoi R package (v1.8.0).^[^ ^116^ ^]^ All analyses have been performed using R 4.2.1. Genes with an adjusted p‐value less than 0.05 were considered to be significant.

### Statistical Analysis

All statistical analyses were performed using R (v4.3.1), unless otherwise specified. The specific packages were detailed through the Experimental section. Information on statistical tests and sample sizes can be found in the relevant subsections.

### Ethics Approval and Patient Consent Statement

The animal experiments conducted in this study were approved by the Animal Care Committee of the University of Santiago de Compostela, in accordance with the institutional guidelines and the European Union standards for the care and use of experimental animals. The publicly available human data used in this study was fully anonymized, and did not require any additional ethical approval or informed consent for its use. All data were obtained and analyzed in compliance with the respective data repository's guidelines and ethical regulations. Access to the protected information in GTEx was approved by the relevant review board (application number: #39922).

## Conflict of Interest

The authors declare no conflict of interest.

## Author Contributions

G.S.A, S.J, A.d.S. performed conceptualization. G.S.A, S.J. performed data collection and curation. G.S.A, S.J, J.A.H. performed data analysis. G.S.A. performed platform implementation. C.I, R.N. performed experimental design. C.I. performed experimental work. G.S.A, C.I, S.J. performed visualization. G.S.A, C.I, S.J, R.N, A.d.S. wrote the manuscript. A.d.S, R.N. performed supervision.

## Supporting information



Supporting Information

## Data Availability

This study utilized publicly available human data from TBI and GTEx, which can be accessed at http://www.aging.brain‐map.org and https://www.gtexportal.org/home/downloads/adult‐gtex/bulk_tissue_expression, and data from the RNAseq Harmonization Study (rnaSeqRe‐processing) and the Living Brain Project (LBP) data, available upon request at AD Knowledge Portal (https://www.adknowledgeportal.synapse.org). GTEx metadata with the age details can be accessed upon request. These datasets are fully anonymized and comply with all applicable ethical and legal standards. No additional ethical approval or informed consent was required for its use, as per the datasets's terms of access. The RNA‐seq data generated in this study have been deposited with links to BioProject accession number PRJNA1287059 in the NCBI BioProject database (https://ncbi.nlm.nih.gov/bioproject). The code used for the preprocessing and analysis depicted in this study, as well as for the training of our brain‐specific transcriptomic clock is available in the following repository https://www.gitlab.lcsb.uni.lu/CBG/brain_clock. Our computational platform is available as an R package, brainAgeShiftR, in the following repository https://www.gitlab.lcsb.uni.lu/CBG/brainAgeShiftR.

## References

[1] World Health Organization T. Aging and Health, 2024, https://www.who.int/news‐room/fact‐sheets/detail/ageing‐and‐health (accessed: October 2024).

[2] T. Niccoli , L. Partridge , Curr. Biol. 2012, 22, R741. 22975005 10.1016/j.cub.2012.07.024

[3] GBD 2019 Diseases and Injuries Collaborators , Lancet 2020, 396, 1160.33069325

[4] A. Y. Chang , V. F. Skirbekk , S. Tyrovolas , N. J. Kassebaum , J. L. Dieleman , Lancet Public Health 2019, 4, 159. 10.1016/S2468-2667(19)30019-2 PMC6472541 30851869

[5] J. D. Steinmetz , K. M. Seeher , N. Schiess , E. Nichols , B. Cao , C. Servili , V. Cavallera , E. Cousin , H. Hagins , M. E. Moberg , M. L. Mehlman , Y. H. Abate , J. Abbas , M. A. Abbasi , M. Abbasian , H. Abbastabar , M. Abdelmasseh , M. Abdollahi , M. Abdollahi , M.‐A. Abdollahifar , R. Abd‐Rabu , D. M. Abdulah , A. Abdullahi , A. Abedi , V. Abedi , R. A. Abeldano Zuniga , H. Abidi , O. Abiodun , R. G. Aboagye , H. Abolhassani , et al., Lancet Neurol. 2024, 23, 344.38493795

[6] M. K. Foret , C. Orciani , L. A. Welikovitch , C. Huang , A. C. Cuello , Commun. Biol. 2024, 7, 861 (2024). 39004677 10.1038/s42003-024-06552-4 PMC11247100

[7] A. Salminen , K. Kaarniranta , A. Kauppinen , J. Mol. Med. 2020, 98, 633. 32279085 10.1007/s00109-020-01904-z PMC7220864

[8] E. A. Newcombe , J. Camats‐Perna , M. L. Silva , N. Valmas , T. J. Huat , R. Medeiros , J. Neuroinflammation 2018, 15, 276. 30249283 10.1186/s12974-018-1313-3 PMC6154824

[9] P. T. Nelson , E. Head , F. A. Schmitt , P. R. Davis , J. H. Neltner , G. A. Jicha , E. L. Abner , C. D. Smith , L. J. Van Eldik , R. J. Kryscio , S. W. Scheff , Acta Neuropathol. 2011, 121, 571. 21516511 10.1007/s00401-011-0826-y PMC3179861

[10] A. A. Podtelezhnikov , K. Q. Tanis , M. Nebozhyn , W. J. Ray , D. J. Stone , A. P. Loboda , PLoS One 2011, 6, 29610. 10.1371/journal.pone.0029610 PMC3247273 22216330

[11] S. Christman , C. Bermudez , L. Hao , B. A. Landman , B. Boyd , K. Albert , N. Woodward , S. Shokouhi , J. Vega , P. Andrews , W. D. Taylor , Transl Psychiatry 2020, 10, 317. 32948749 10.1038/s41398-020-01004-z PMC7501280

[12] W.‐C. T. Ageing , Nature 2016, 539, 180. 27830812 10.1038/nature20411 PMC5172605

[13] G. Bieri , A. B. Schroer , S. A. Villeda , Nat. Neurosci. 2023, 26, 379. 36646876 10.1038/s41593-022-01238-8

[14] M. Xu , J. Y. Zhu , X. D. Liu , M. Y. Luo , N. J. Xu , Transl Neurodegener 2021, 10, 2021. 10.1186/s40035-021-00254-1 PMC8361623 34389067

[15] A. Herring , Y. Münster , J. Metzdorf , B. Bolczek , S. Krüssel , D. Krieter , I. Yavuz , F. Karim , C. Roggendorf , A. Stang , Y. Wang , D. M. Hermann , S. Teuber‐Hanselmann , K. Keyvani , Neurobiol Dis 2016, 94, 44. 27312772 10.1016/j.nbd.2016.06.003

[16] L. Katsimpardi , N. K. Litterman , P. A. Schein , C. M. Miller , F. S. Loffredo , G. R. Wojtkiewicz , J. W. Chen , R. T. Lee , A. J. Wagers , L. L. Rubin , Science 1979, 344, 630. 10.1126/science.1251141 PMC4123747 24797482

[17] S. A. Villeda , K. E. Plambeck , J. Middeldorp , J. M. Castellano , K. I. Mosher , J. Luo , L. K. Smith , G. Bieri , K. Lin , D. Berdnik , R. Wabl , J. Udeochu , E. G. Wheatley , B. Zou , D. A. Simmons , X. S. Xie , F. M. Longo , T. Wyss‐Coray , Nat. Med. 2014, 20, 659. 24793238 10.1038/nm.3569 PMC4224436

[18] B. M. Brown , J. Peiffer , S. R. Rainey‐Smith , Ageing Res. Rev. 2018, 50, 9. 10.1016/j.arr.2019.01.003 30615936

[19] S. Gillette‐Guyonnet , B. Vellas , Curr. Opin. Clin. Nutr. Metab. Care 2008, 11, 686. 18827571 10.1097/MCO.0b013e328313968f

[20] P. Zhang , Y. Kishimoto , I. Grammatikakis , K. Gottimukkala , R. G. Cutler , S. Zhang , K. Abdelmohsen , V. A. Bohr , J. Misra Sen , M. Gorospe , M. P. Mattson , Nat. Neurosci. 2019, 22, 719. 30936558 10.1038/s41593-019-0372-9 PMC6605052

[21] R. S. Turner , R. G. Thomas , S. Craft , C. H. van Dyck , J. Mintzer , B. A. Reynolds , J. B. Brewer , R. A. Rissman , R. Raman , P. S. Aisen , J. Mintzer , B. A. Reynolds , J. Karlawish , D. Galasko , J. Heidebrink , N. Aggarwal , N. Graff‐Radford , M. Sano , R. Petersen , K. Bell , R. Doody , A. Smith , C. Bernick , A. Porteinsson , P. Tariot , R. Mulnard , A. Lerner , L. Schneider , J. Burns , M. Raskind , et al., Neurology 2015, 85, 1383. 26362286 10.1212/WNL.0000000000002035 PMC4626244

[22] M. Ximerakis , K. M. Holton , R. M. Giadone , C. Ozek , M. Saxena , S. Santiago , X. Adiconis , D. Dionne , L. Nguyen , K. M. Shah , J. M. Goldstein , C. Gasperini , I. A. Gampierakis , S. L. Lipnick , S. K. Simmons , S. M. Buchanan , A. J. Wagers , A. Regev , J. Z. Levin , L. L. Rubin , Nat. Aging. 2023, 3, 327. 37118429 10.1038/s43587-023-00373-6 PMC10154248

[23] S. L. McGee , H. M. Epigenetics , Trends in Endocrinol. Metabolism. 2019, 30, 636. 10.1016/j.tem.2019.06.002 31279665

[24] V. Izquierdo , V. Palomera‐ávalos , M. Pallàs , C. Griñán‐Ferré , Int. J. Mol. Sci. 2021, 22, 1453. 33535619 10.3390/ijms22031453 PMC7867164

[25] K. C. McGee , J. Sullivan , J. Hazeldine , L. J. Schmunk , D. E. Martin‐Herranz , T. Jackson , J. M. Lord , Geroscience 2024, 46, 4333. 38528176 10.1007/s11357-024-01138-8 PMC11336001

[26] E. Lee , N. Carreras‐Gallo , L. Lopez , L. Turner , A. Lin , T. L. Mendez , H. Went , A. Tomusiak , E. Verdin , M. Corley , L. Ndhlovu , R. Smith , V. B. Dwaraka , Aging 2024, 16, 3088. 38393697 10.18632/aging.205581 PMC10929829

[27] Y. Shen , S. Zaballa , X. Bech , A. Sancho‐Balsells , I. Rodriguez‐Navarro , C. Cifuentes‐Diaz , G. Seyit‐Bremer , S. H. Chun , T. Straub , J. Abante , I. Merino‐Valverde , L. Richart , V. Gupta , H. Li , Cell Stem Cell 2024, 31, 1741. 39426381 10.1016/j.stem.2024.09.013

[28] A. Huyghe , A. Trajkova , F. Lavial , Trends Cell Biol. 2024, 34, 255. 37648593 10.1016/j.tcb.2023.07.013

[29] A. P. Singh , R. Singh , S. S. Verma , V. Rai , C. H. Kaschula , P. Maiti , S. C. Gupta , Med. Res. Reviews 2019, 39, 1851. 10.1002/med.21565 30741437

[30] J. J. Martínez‐Magaña , J. Hurtado‐Soriano , N. A. Rivero‐Segura , J. L. Montalvo‐Ortiz , P. Garcia‐delaTorre , K. Becerril‐Rojas , J. C. Gomez‐Verjan , Arch. Med. Res. 2024, 55, 135203033. 10.1016/j.arcmed.2024.103033 38955096

[31] L. Kananen , S. Marttila , T. Nevalainen , L. Kummola , I. Junttila , N. Mononen , M. Kähönen , O. T. Raitakari , A. Hervonen , M. Jylhä , T. Lehtimäki , M. Hurme , J. Jylhävä , Age (Omaha) 2016, 38, 1741. 10.1007/s11357-016-9927-9 PMC5005919 27300324

[32] J. Jylhävä , N. L. Pedersen , H. S. B. A. Predictors , EBioMedicine. 2017, 21, 29. 28396265 10.1016/j.ebiom.2017.03.046 PMC5514388

[33] C. G. Bell , R. Lowe , P. D. Adams , A. A. Baccarelli , S. Beck , J. T. Bell , B. C. Christensen , V. N. Gladyshev , B. T. Heijmans , S. Horvath , T. Ideker , J.‐P. J. Issa , K. T. Kelsey , R. E. Marioni , W. Reik , C. L. Relton , L. C. Schalkwyk , A. E. Teschendorff , W. Wagner , K. Zhang , V. K. Rakyan , Genome Biol. 2019, 20, 249 (2019). 31767039 10.1186/s13059-019-1824-y PMC6876109

[34] M. E. Levine , A. T. Lu , A. Quach , B. H. Chen , T. L. Assimes , S. Bandinelli , L. Hou , A. A. Baccarelli , J. D. Stewart , Y. Li , E. A. Whitsel , J. G. Wilson , A. P. Reiner , A. Aviv , K. Lohman , Y. Liu , L. Ferrucci , S. Horvath , Aging 2018, 10, 573. 29676998 10.18632/aging.101414 PMC5940111

[35] K. L. Thrush , D. A. Bennett , C. Gaiteri , S. Horvath , C. H. V. Dyck , A. T. Higgins‐Chen , M. E. Levine , Aging 2022, 14, 5641. 35907208 10.18632/aging.204196 PMC9365556

[36] F. Grodstein , B. Lemos , L. Yu , H.‐U. Klein , A. Iatrou , A. S. Buchman , G. L. Shireby , J. Mill , J. A. Schneider , P. L. De Jager , D. A. Bennett , Neurobiol. Dis. 2022, 157, 105428. 10.1016/j.nbd.2021.105428 PMC8373772 34153464

[37] A. M. Plesa , S. Jung , H. H. Wang , F. Omar , M. Shadpour , D. C. Buentello , M. C. Perez‐Matos , N. Horwitz , G. Cai , Z. Ngian , C. V. de Magalhaes , A. J. Wagers , W. B. Mair , A. del Sol , G. M. Church , bioRxiv 2023, 45, 580.

[38] R. J. Hodes , N. Buckholtz , Expert Opin. Ther. Targets 2016, 20, 389. 26853544 10.1517/14728222.2016.1135132

[39] J. Lonsdale , J. Thomas , M. Salvatore , R. Phillips , E. Lo , S. Shad , R. Hasz , G. Walters , F. Garcia , N. Young , B. Foster , M. Moser , E. Karasik , B. Gillard , K. Ramsey , S. Sullivan , J. Bridge , H. Magazine , J. Syron , J. Fleming , L. Siminoff , H. Traino , M. Mosavel , L. Barker , S. Jewell , D. Rohrer , D. Maxim , D. Filkins , P. Harbach , E. Cortadillo , et al., Nat. Genet. 2013, 45, 580. 23715323 10.1038/ng.2653 PMC4010069

[40] J. A. Miller , A. Guillozet‐Bongaarts , L. E. Gibbons , N. Postupna , A. Renz , A. E. Beller , S. M. Sunkin , L. Ng , S. E. Rose , K. A. Smith , A. Szafer , C. Barber , D. Bertagnolli , K. Bickley , K. Brouner , S. Caldejon , M. Chapin , M. L. Chua , N. M. Coleman , E. Cudaback , C. Cuhaciyan , R. A. Dalley , N. Dee , T. Desta , T. A. Dolbeare , N. I. Dotson , M. Fisher , N. Gaudreault , G. Gee , T. L. Gilbert , et al., Elife 2017, 6, 31126. 10.7554/eLife.31126 PMC5679757 29120328

[41] L. Collado‐Torres , E. E. Burke , A. Peterson , J. Shin , R. E. Straub , A. Rajpurohit , S. A. Semick , W. S. Ulrich , A. J. Price , C. Valencia , R. Tao , A. Deep‐Soboslay , T. M. Hyde , J. E. Kleinman , D. R. Weinberger , A. E. Jaffe , Neuron 2019, 103, 203. 31174959 10.1016/j.neuron.2019.05.013 PMC7000204

[42] A. E. Jaffe , R. E. Straub , J. H. Shin , R. Tao , Y. Gao , L. Collado‐Torres , T. Kam‐Thong , H. S. Xi , J. Quan , Q. Chen , C. Colantuoni , W. S. Ulrich , B. J. Maher , A. Deep‐Soboslay , A. J. Cross , N. J. Brandon , J. T. Leek , T. M. Hyde , J. E. Kleinman , D. R. Weinberger , Nat. Neurosci. 2018, 21, 1117. 30050107 10.1038/s41593-018-0197-y PMC6438700

[43] M. Signorelli , V. Vinciotti , E. C. Wit , BMC Bioinformatics 2016, 17, 2016. 10.1186/s12859-016-1203-6 PMC5011912 27597310

[44] G. Saher , Annu. Rev. Neurosci. 2023, 10, 59. 10.1146/annurev-neuro-091922-034237 37428605

[45] K. Huang , H. Gong , J. Guan , L. Zhang , C. Hu , W. Zhao , L. Huang , W. Zhang , P. Kim , X. Zhou , Nucleic Acids Res. 2023, 51, 580. 10.1093/nar/gkac847 PMC9825500 36200838

[46] A. Subramanian , R. Narayan , S. M. Corsello , D. D. Peck , T. E. Natoli , X. Lu , J. Gould , J. F. Davis , A. A. Tubelli , J. K. Asiedu , D. L. Lahr , J. E. Hirschman , Z. Liu , M. Donahue , B. Julian , M. Khan , D. Wadden , I. C. Smith , D. Lam , A. Liberzon , C. Toder , M. Bagul , M. Orzechowski , O. M. Enache , F. Piccioni , S. A. Johnson , N. J. Lyons , A. H. Berger , A. F. Shamji , A. N. Brooks , et al., Cell 2017, 171, 1437e17. 29195078 10.1016/j.cell.2017.10.049 PMC5990023

[47] H. Minami , Y. Fujiwara , K. Muro , M. Sato , A. Moriya , Cancer Chemother. Pharmacol. 2019, 84, 337. 31190275 10.1007/s00280-019-03883-6

[48] D. J. W. Lee , A. H. Kuerec , A. B. Maier , Lancet Healthy Longev 2024, 5, 152. 10.1016/S2666-7568(23)00258-1 38310895

[49] C. V. Rao , A. S. Asch , D. J. J. Carr , H. Y. Yamada , Aging Cell 2019, 19, 13109. 10.1111/acel.13109 PMC7059149 31981470

[50] K. Yu , L. Toral‐Barza , C. Shi , W.‐G. Zhang , J. Lucas , B. Shor , J. Kim , J. Verheijen , K. Curran , D. J. Malwitz , D. C. Cole , J. Ellingboe , S. Ayral‐Kaloustian , T. S. Mansour , J. J. Gibbons , R. T. Abraham , P. Nowak , A. Zask , Cancer Res. 2009, 69, 6232. 19584280 10.1158/0008-5472.CAN-09-0299

[51] H. H. Leuchte , J. Behr , Expert Review in Cardiovascuolar Therapy 2005, 3, 215. 10.1586/14779072.3.2.215 15853595

[52] R. Du , C. Huang , K. Liu , X. Li , Z. Dong , Mol. Cancer 2021, 20, 15. 33451333 10.1186/s12943-020-01305-3 PMC7809767

[53] M. R. Baer , C. Gambacorti‐passerini , J. Mccloskey , Y. Minami , C. Papayannidis , JAMA, J. Am. Med. Assoc. 2024, 331, 1814. 10.1001/jama.2024.4783 PMC11082750 38722621

[54] H. Xu , M. Di Antonio , S. McKinney , V. Mathew , B. Ho , N. J. O'Neil , N. D. Santos , J. Silvester , V. Wei , J. Garcia , F. Kabeer , D. Lai , P. Soriano , J. Banáth , D. S. Chiu , D. Yap , D. D. Le , F. B. Ye , A. Zhang , K. Thu , J. Soong , S.‐C. Lin , A. H. C. Tsai , T. Osako , T. Algara , D. N. Saunders , J. Wong , J. Xian , M. B. Bally , J. D. Brenton , et al., Nat. Commun. 2017, 8, 14432. 28211448 10.1038/ncomms14432 PMC5321743

[55] S. D. Zabludoff , C. Deng , M. R. Grondine , A. M. Sheehy , S. Ashwell , B. L. Caleb , S. Green , H. R. Haye , C. L. Horn , J. W. Janetka , D. Liu , E. Mouchet , S. Ready , J. L. Rosenthal , C. Queva , G. K. Schwartz , K. J. Taylor , A. N. Tse , G. E. Walker , A. M. White , Mol. Cancer Ther. 2008, 7, 2955. 18790776 10.1158/1535-7163.MCT-08-0492

[56] X. Zhao , W. Sun , W. M. Puszyk , S. Wallet , S. Hochwald , K. Robertson , C. Liu , Tumor Biology. 2017, 39, 1010428317699120 28459212 10.1177/1010428317699120

[57] D. Barardo , D. Thornton , H. Thoppil , M. Walsh , S. Sharifi , S. Ferreira , A. Anzic , M. Fernandes , P. Monteiro , T. Grum , R. Cordeiro , E. A. De‐Souza , A. Budovsky , N. Araujo , J. Gruber , M. Petrascheck , V. E. Fraifeld , A. Zhavoronkov , A. Moskalev , J. P. de Magalhães , Aging Cell 2017, 16, 594. 28299908 10.1111/acel.12585 PMC5418190

[58] F. Matteini , S. Montserrat‐Vazquez , M. C. Florian , FEBS Lett. 2024, 598, 2776. 38604982 10.1002/1873-3468.14865 PMC11586596

[59] C. López‐Otín , M. A. Blasco , L. Partridge , M. Serrano , G. Kroemer , Cell 2013, 153, 1194. 23746838 10.1016/j.cell.2013.05.039 PMC3836174

[60] S. Di Benedetto , L. Müller , E. Wenger , S. Düzel , G. Pawelec , Neurosci Biobehav Rev. 2017, 75, 114. 28161508 10.1016/j.neubiorev.2017.01.044

[61] N. Vitorakis , C. Piperi , Int. J. Mol. Sci. 2023, 24, 7339. 38139167 10.3390/ijms242417339 PMC10744334

[62] K. Wang , H. Liu , Q. Hu , L. Wang , J. Liu , Z. Zheng , W. Zhang , J. Ren , F. Zhu , G. Liu , Signal Transduct Target Ther. 2022, 7, 374. 36336680 10.1038/s41392-022-01211-8 PMC9637765

[63] F. Sesti , A. Bortolami , in Factors Affecting Neurological Aging (Eds: C R. Martin , R. Victor , R. R. Preedy ), Academic Press, New York, NY 2021, pp. 237‐245.

[64] Y. C. Martin , J. L. Kofron , L. M. Traphagen , J. Med. Chem. 2002, 45, 4350. 12213076 10.1021/jm020155c

[65] F. Caraci , G. Pappalardo , L. Basile , A. Giuffrida , A. Copani , R. Tosto , A. Sinopoli , M. L. Giuffrida , E. Pirrone , F. Drago , R. Pignatello , S. Guccione , Eur. J. Pharmacol. 2015, 764, 256. 26162702 10.1016/j.ejphar.2015.07.015

[66] H. Park , K.‐M. Han , H. Jeon , J.‐S. Lee , H. Lee , S. G. Jeon , J.‐H. Park , Y. G. Kim , Y. Lin , Y.‐H. Lee , Y. H. Jeong , H.‐S. Hoe , Cells 2020, 9, 1982.32872335

[67] A. Y. Berman , R. A. Motechin , M. Y. Wiesenfeld , M. K. Holz , NPJ Precis Oncol. 2017, 1, 35. 28989978 10.1038/s41698-017-0038-6 PMC5630227

[68] A. Zia , T. Farkhondeh , P.‐A. M. , S. Samarghandian , Biomed. Pharmacother. 2021, 134, 111119. 33360051 10.1016/j.biopha.2020.111119

[69] C. Niederau , S. Bhargava , R. S. Kramman , J. Jankowski , R. B. Craveiro , M. Wolf , Sci Rep. 2022, 14970. 36056072 10.1038/s41598-022-19220-6 PMC9440237

[70] Y. Zhou , Y. Zhang , D. Zhao , X. Yu , X. Shen , Y. Zhou , S. Wang , Y. Qiu , Y. Chen , F. Zhu , Nucleic Acids Res. 2024, 52, D1465. 37713619 10.1093/nar/gkad751 PMC10767903

[71] J. Guan , G. Wang , J. Wang , Z. Zhang , Y. Fu , L. Cheng , G. Meng , Y. Lyu , J. Zhu , Y. Li , Y. Wang , S. Liuyang , B. Liu , Z. Yang , H. He , X. Zhong , Q. Chen , X. Zhang , S. Sun , W. Lai , Y. Shi , L. Liu , L. Wang , C. Li , S. Lu , H. Deng , Nature 2022, 605, 325. 35418683 10.1038/s41586-022-04593-5

[72] R. M. Angell , F. L. Atkinson , M. J. Brown , T. T. Chuang , J. A. Christopher , M. Cichy‐Knight , A. K. Dunn , K. E. Hightower , S. Malkakorpi , J. R. Musgrave , M. Neu , P. Rowland , R. L. Shea , J. L. Smith , D. O. Somers , S. A. Thomas , G. Thompson , R. Wang , Bioorg. Med. Chem. Lett. 2007, 17, 1296. 17194588 10.1016/j.bmcl.2006.12.003

[73] K. S. Abdelrahman , H. A. Hassan , S. A. Abdel‐Aziz , A. A. Marzouk , A. Narumi , H. Konno , M. Abdel‐Aziz , Pharmacol. Rep. 2021, 73, 405. 33710509 10.1007/s43440-021-00238-y

[74] L. Prut , C. Belzung , Eur. J. Pharmacol. 2003, 463, 3. 12600700 10.1016/s0014-2999(03)01272-x

[75] L. M. Lueptow , J. Visualized Experiments 2017, e55718. 10.3791/55718 PMC5614391 28892027

[76] A. Liberzon , C. Birger , H. Thorvaldsdóttir , M. Ghandi , J. P. Mesirov , P. Tamayo , Cell Syst 2015, 1, 417. 26771021 10.1016/j.cels.2015.12.004 PMC4707969

[77] T. Tabula , M. Consortium , Nature 2020, 583, 590.32669714

[78] J. J. Martínez‐Magaña , J. H. Krystal , M. J. Girgenti , D. L. Nunez‐Rios , S. T. Nagamatsu , D. E. Andrade‐Brito , medRxiv 2023, 23288765.

[79] X. Ren , P. F. Kuan , PLoS One 2020, 15, 0237006. 10.1371/journal.pone.0237006 PMC7402472 32750074

[80] H. Qi , H. Zhao , E. Li , X. Lu , N. Yu , J. Liu , J. Han , Aging Cell 2025, 24, 14471. 10.1111/acel.14471 PMC12074024 39757434

[81] H. M. Chow , K. Herrup , Nat. Rev. Neurosci. 2015, 16, 672. 26462757 10.1038/nrn4020

[82] M. T. Buckley , E. D. Sun , B. M. George , L. Liu , N. Schaum , L. Xu , J. M. Reyes , M. A. Goodell , I. L. Weissman , T. Wyss‐Coray , T. A. Rando , A. Brunet , Nat. Aging 2023, 3, 121. 37118510 10.1038/s43587-022-00335-4 PMC10154228

[83] P. V. Kharchenko , L. Silberstein , D. T. Scadden , Nat. Methods 2014, 11, 740. 24836921 10.1038/nmeth.2967 PMC4112276

[84] A. Elobeid , S. Libard , M. Leino , S. N. Popova , I. Alafuzoff , J. Neuropathol Exp. Neurol. 2016, 75, 316. 26979082 10.1093/jnen/nlw002 PMC4793886

[85] R. E. Mrak , W. S. T. Griffin , D. I. Graham , J Neuropathol Exp Neurol. 1997, 56, 1269. 9413275 10.1097/00005072-199712000-00001

[86] S. Horvath , Genome Biol. 2013, 14, R115. 24138928 10.1186/gb-2013-14-10-r115 PMC4015143

[87] A. Leparulo , M. Bisio , N. Redolfi , T. Pozzan , S. Vassanelli , C. Fasolato , Cells 2022, 11, 238. 35053352 10.3390/cells11020238 PMC8774248

[88] S. Ji , M. Xiong , H. Chen , Y. Liu , L. Zhou , Y. Hong , M. Wang , C. Wang , X. Fu , X. Sun , Signal Transduct Target Ther 2023, 8, 116. 36918530 10.1038/s41392-023-01343-5 PMC10015098

[89] K. Jin , Z. Yao , C. T. J. van Velthoven , E. S. Kaplan , K. Glattfelder , S. T. Barlow , G. Boyer , D. Carey , T. Casper , A. B. Chakka , R. Chakrabarty , M. Clark , M. Departee , M. Desierto , A. Gary , J. Gloe , J. Goldy , N. Guilford , J. Guzman , D. Hirschstein , C. Lee , E. Liang , T. Pham , M. Reding , K. Ronellenfitch , A. Ruiz , J. Sevigny , N. Shapovalova , L. Shulga , J. Sulc , bioRxiv 2023, 550355.

[90] X. Wen , Z. Luo , W. Zhao , R. Calandrelli , T. C. Nguyen , X. Wan , J. L. Charles Richard , S. Zhong , Nature 2024, 628, 648. 38538789 10.1038/s41586-024-07239-w PMC11023937

[91] J.‐F. Chien , H. Liu , B.‐A. Wang , C. Luo , A. Bartlett , R. Castanon , N. D. Johnson , J. R. Nery , J. Osteen , J. Li , J. Altshul , M. Kenworthy , C. Valadon , M. Liem , N. Claffey , C. O'Connor , L. A. Seeker , J. R. Ecker , M. M. Behrens , E. A. Mukamel , Neuron 2024, 112, 2524 38838671 10.1016/j.neuron.2024.05.013

[92] Y. Kim , J. S. Lee , Y. H. Joo , Transl Psychiatry 2020, 10, 156. 32424120 10.1038/s41398-020-0838-2 PMC7235015

[93] X. Zhang , X. He , Q. Li , X. Kong , Z. Ou , L. Zhang , Z. Gong , D. Long , J. Li , M. Zhang , W. Ji , W. Zhang , L. Xu , A. Xuan , Stem Cell Rep. 2017, 8, 1256. 10.1016/j.stemcr.2017.04.006 PMC5425725 28494938

[94] A. E. Budson , B. H. Price , N. Engl. J. Med. 2005, 352, 692. 15716563 10.1056/NEJMra041071

[95] X. Gao , T. Geng , M. Jiang , N. Huang , Y. Zheng , D. W. Belsky , T. Huang , Nat. Commun. 2023, 14, 2277. 37080981 10.1038/s41467-023-38013-7 PMC10119095

[96] B. Pereira , F. P. Correia Alves , I. A. Alves , M. Costa , M. Gameiro , A. P. Martins , J. A. Saraiva , Ageing Res. Rev. 2024, 95, 1568. 10.1016/j.arr.2024.102204 38272265

[97] J. A. Goldman , K. D. Poss , Nat. Rev. Genet. 2020, 21, 511. 32504079 10.1038/s41576-020-0239-7 PMC7448550

[98] C. Jopling , S. Boue , J. C. I. Belmonte , Nat. Rev. Mol. Cell Biol. 2011, 12, 79. 21252997 10.1038/nrm3043

[99] A. D. Yücel , V. N. Gladyshev , Nat. Commun. 2024, 15, 1941. 38431638 10.1038/s41467-024-46020-5 PMC10908844

[100] S. Deng , H. Xie , B. Xie , Stem. Cell Res. Ther. 2025, 16, 176. 40189500 10.1186/s13287-025-04285-7 PMC11974143

[101] D. A. Bennett , A. S. Bichman , P. A. Boyle , L. L. Barnes , R. S. Wilson , J. A. Schneider , J. Alzheimers Disease 2018, 64, S161. 29865057 10.3233/JAD-179939 PMC6380522

[102] M. Allen , M. M. Carrasquillo , C. Funk , B. D. Heavner , F. Zou , C. S. Younkin , J. D. Burgess , H.‐S. Chai , J. Crook , J. A. Eddy , H. Li , B. Logsdon , M. A. Peters , K. K. Dang , X. Wang , D. Serie , C. Wang , T. Nguyen , S. Lincoln , K. Malphrus , G. Bisceglio , M.a Li , T. E. Golde , L. M. Mangravite , Y. Asmann , N. D. Price , R. C. Petersen , N. R. Graff‐Radford , D. W. Dickson , S. G. Younkin , et al., Sci. Data 2016, 3, 160089. 27727239 10.1038/sdata.2016.89 PMC5058336

[103] M. Wang , N. D. Beckmann , P. Roussos , E. Wang , X. Zhou , Q. Wang , C. Ming , R. Neff , W. Ma , J. F. Fullard , M. E. Hauberg , J. Bendl , M. A. Peters , B. Logsdon , P. Wang , M. Mahajan , L. M. Mangravite , E. B. Dammer , D. M. Duong , J. J. Lah , N. T. Seyfried , A. I. Levey , J. D. Buxbaum , M. Ehrlich , S. Gandy , P. Katsel , V. Haroutunian , E. Schadt , B. Zhang , Sci. Data 2018, 5, 180185. 30204156 10.1038/sdata.2018.185 PMC6132187

[104] Y. Hao , T. Stuart , M. H. Kowalski , S. Choudhary , P. Hoffman , A. Hartman , A. Srivastava , G. Molla , S. Madad , C. Fernandez‐Granda , R. Satija , Nat. Biotechnol. 2024, 42, 293. 37231261 10.1038/s41587-023-01767-y PMC10928517

[105] C. S. McGinnis , L. M. Murrow , G. Z. J. DoubletFinder , Cell Syst 2019, 8, 329. 30954475 10.1016/j.cels.2019.03.003 PMC6853612

[106] J. Chen , E. Behnam , J. Huang , M. F. Moffatt , D. J. Schaid , L. Liang , X. Lin , BMC Genomics 2017, 18, 413 (2017). 28549425 10.1186/s12864-017-3808-1 PMC5446715

[107] J. T. Leek , W. E. Johnson , H. S. Parker , A. E. Jaffe , J. D. Storey , Bioinformatics 2012, 28, 882. 22257669 10.1093/bioinformatics/bts034 PMC3307112

[108] T. Fryda , E. LeDell , N. Gill , S. Aiello , A. Fu , A. Candel , C. Click , T. Kraljevic , T. Nykodym , P. Aboyoun , M. Kurka , M. Malohlava , S. Poirier , W. Wong , h2o, R Interface for the “H2O” Scalable Machine Learning Platform R package version 3.46.0.2, 2024, https://www.github.com/h2oai/h2o‐3 (accessed: August 2024).

[109] E. Persson , M. Castresana‐Aguirre , D. Buzzao , D. Guala , E. L. L. Sonnhammer , J. Mol. Biol. 2021, 433, 166835. 33539890 10.1016/j.jmb.2021.166835

[110] M. Carlson , org.Hs.eg.db: Genome wide annotation for Human. R package version 3.17.0. Published online 2023.

[111] Y. Cao , A. Charisi , L. C. Cheng , T. Jiang , T. Girke , Bioinformatics 2008, 24, 1733. 18596077 10.1093/bioinformatics/btn307 PMC2638865

[112] A. Kassambara, rstatix : Pipe‐Friendly Framework for Basic Statistical Tests R package version 0.7.2, 2023, https://www.cran.r‐project.org/package=rstatix (accessed: October 2024).

[113] A. Dobin , C. A. Davis , F. Schlesinger , J. Drenkow , C. Zaleski , S. Jha , P. Batut , M. Chaisson , T. R. Gingeras , Bioinformatics 2013, 29, 15. 23104886 10.1093/bioinformatics/bts635 PMC3530905

[114] Y. Liao , G. K. Smyth , S. W. FeatureCounts , Bioinformatics 2014, 30, 923. 24227677 10.1093/bioinformatics/btt656

[115] M. I. Love , W. Huber , S. Anders , Genome Biol. 2014, 15, 550. 25516281 10.1186/s13059-014-0550-8 PMC4302049

[116] C. Ahlmann‐Eltze , W. Huber , Bioinformatics 2020, 36, 5701. 10.1093/bioinformatics/btaa1009 PMC8023675 33295604

