# Supplementary material for: A Machine‐Learning Approach Identifies Rejuvenating Interventions in the Human Brain
Source: Adv Sci (Weinh). 2025 Jul 14;12(37):e03344. doi: 10.1002/advs.202503344 (PMC12499397; doi:10.1002/advs.202503344)
Supplement: Supplementary file 1 — Supporting Information [file ADVS-12-e03344-s001.docx]

# Supplementary Material

### Supplementary figures

Figure S1. Performance of our human brain-specific transcriptomic clock in different subsets of the internal test set (1/3 held out from the healthy controls). A. Chronological age distribution of the samples that belong to each one of the substudies we used in our study. B. Coefficient of determination (R^2^) per substudy. C. Mean absolute error (MAE) per susbtudy. D. Chronological age distribution of the samples per sex. E. R^2^ per sex. F. MAE per sex. G. Relationship between R^2^ and standard deviation of the samples that originate from each one of the brain regions used in our study. Each point corresponds to a brain region. H. Relationship between MAE and standard deviation of the samples that originate from each one of the brain regions used in our study. Each point corresponds to a brain region.

Figure S2. Treemap plot of the GO biological processes enriched in the genes that were used by our brain-specific transcriptional clock to predict age. The enrichment was performed with Network Enrichment Analysis Test (NEAT), using *H. sapiens* FunCoup 5.0, considering only the links that had a confidence index equal or higher than 0.75.

Figure S3. Treemap plot of the GO molecular functions enriched in the genes that were used by our brain-specific transcriptional clock to predict age. The enrichment was performed with Network Enrichment Analysis Test (NEAT), using *H. sapiens* FunCoup 5.0, considering only the links that had a confidence index equal or higher than 0.75.

Figure S4. Treemap plot of the GO cellular components enriched in the genes that were used by our brain-specific transcriptional clock to predict age. The enrichment was performed with Network Enrichment Analysis Test (NEAT), using *H. sapiens* FunCoup 5.0, considering only the links that had a confidence index equal or higher than 0.75.

Figure S5. Enriched GO terms, p-value based, in the genes used by our brain-specific transcriptional clock to predict age. The enrichment was performed with Network Enrichment Analysis Test (NEAT), using *H. sapiens* FunCoup 5.0, considering only the links that had a confidence index equal or higher than 0.75. A. Molecular functions. B. Cellular components.

Figure S6. Relationship between chronological and transcriptional age for the pseudo-bulk samples of each one of the cell types. P-values of the corresponding F-tests are showed over each plot, as well as the coefficient of determination (r^2^) and the equation of the fitted model.

Figure S7. Linear models showing the contribution of Braak index to the adjusted transcriptional age, on each decade. The transcriptional age was adjusted by regressing out the chronological age, to account for potential confounding effects. The p-value of the Braak index’s association to the predicted age (F-test) is showed in each plot.


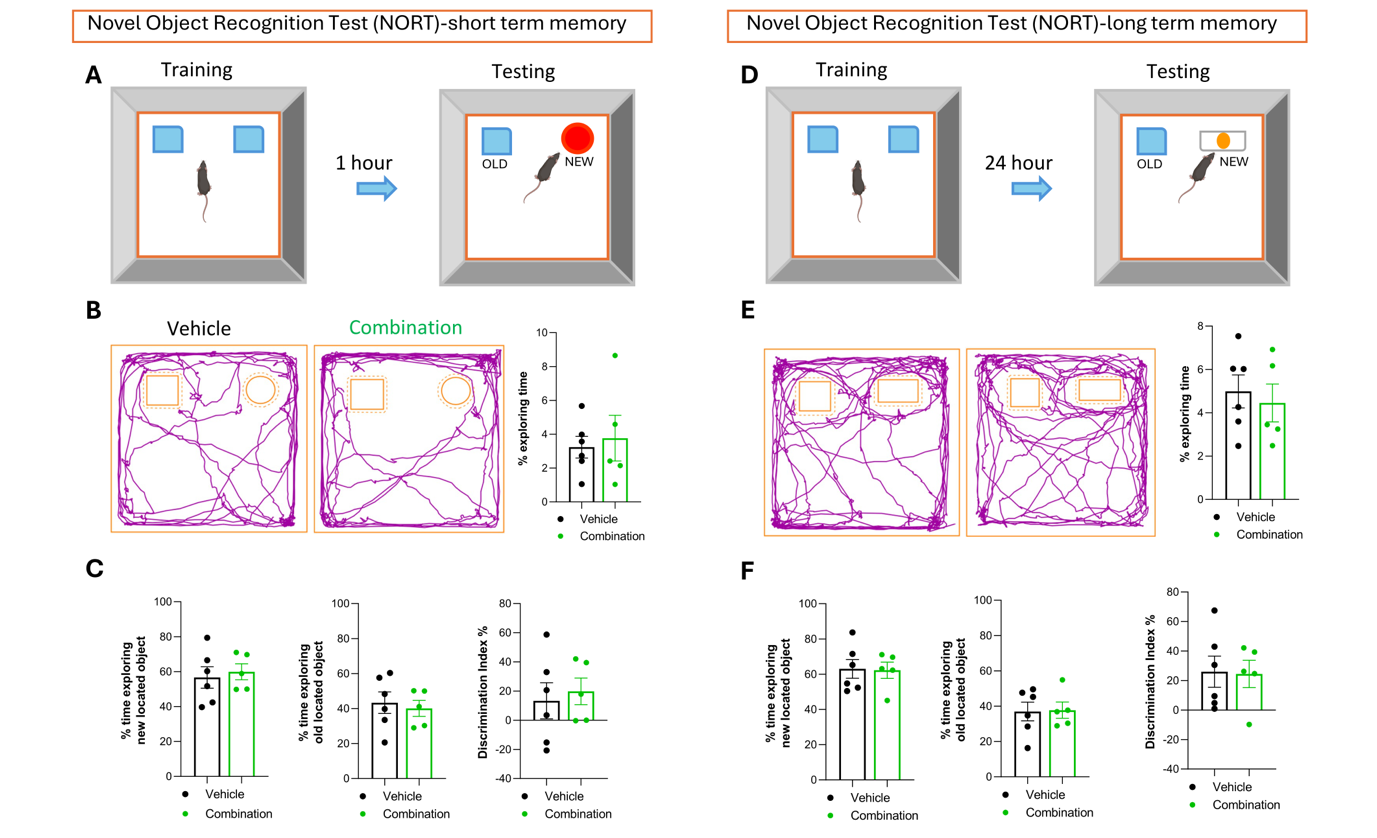


Figure S8. Old mice injected with the combination of three compounds perform similarly to vehicle-treated mice in Novel Object Recognition Tests assessing short- or long-term memory. A/D. Schemes of the Novel Object Recognition Tests (NORT) for short-term and long-term memory, respectively, showing the objects during the training period and the exchange of one object during the testing period. B/E. Track plots of the NORT (short-term and long-term memory, respectively), showing the layout of the animals. The percentage of exploring time was calculated by dividing the time the animals spent exploring the new object by the total duration of the test. C/F. Graphs comparing the percentage of time spent exploring the new object at 1 hour (short-term) or 24 hours (long-term memory) after the training period, in both vehicle- and treated animals. n=6 vehicle-injected animals (DMSO) and n=5 animals injected with the three-compound combination (5-azacytidine, tranylcypromine, and JNK-IN-8), administered intraperitoneally 8 times. *p<0.05, **p<0.01, Student’s t-test.

Figure S9. Cortex differentially expressed genes between old mice treated with the three-compound combination (5-azacytidine, tranylcypromine and JNK-IN-8), and old mice treated with the vehicle. Bulk cortical transcriptome was sequenced with Illumina, and the differentially expressed genes were identified through a Wald test, using DESeq2 (adjusted p-value ≤ 0.05).

### Supplementary tables

Table S1. Genes used by our brain-specific transcriptional clock. The gene symbols, gene names, coefficients and standardized coefficients are indicated, as well as if the genes are related to any brain-specific process.

Table S2. Brain-related transcriptional age predictors.

Table S3. NEAT enriched GO biological processes. The criteria for significant enrichment were adjusted p-value ≤ 0.05 and number of links between set and term (NAB) / expected NAB > 1.

Table S4. NEAT enriched GO molecular functions. The criteria for significant enrichment were adjusted p-value ≤ 0.05 and number of links between set and term (NAB) / expected NAB > 1.

Table S5. NEAT enriched GO cellular components. The criteria for significant enrichment were adjusted p-value ≤ 0.05 and number of links between set and term (NAB) / expected NAB > 1.

Table S6. GEO accessions of the perturbation data that did not originate in LINCS L1000.

Table S7. Perturbations that produced significant rejuvenation in neural progenitor cells (NPCs). Significance is assessed by FDR ≤ 0.05 and delta chronological age (treated minus untreated) < 0. Samples were considered to be subjected to the same perturbation if they shared compound, dose and exposure time, for chemical perturbations, or target gene and perturbation type, for genetic perturbations.

Table S8. Perturbations that produced significant rejuvenation in neurons. Significance is assessed by FDR ≤ 0.05 and delta chronological age (treated minus untreated) < 0. Samples were considered to be subjected to the same perturbation if they shared compound, dose and exposure time, for chemical perturbations, or target gene and perturbation type, for genetic perturbations.

Table S9. Compounds that produced statistically significant rejuvenation in NPCs. The targets, mechanisms of action, SMILES and InChI keys are indicated when available.

Table S10. Compounds that produced statistically significant rejuvenation in neurons. The targets, mechanisms of action, SMILES and InChI keys are indicated when available.

Table S11. Enriched gene sets from MSigDB, determined by NEAT using differentially under-expressed genes between old mice treated with the three-compound combination (5-azacytidine, tranylcypromine and JNK-IN-8), and old mice treated with the vehicle. The level of significance for considering a gene set enrichment was FDR ≤ 0.05, , and NAB/expected NAB > 1, which reflects that the differentially expressed genes are over-represented in the reference set.

Table S12. Enriched gene sets from MSigDB, determined by NEAT using differentially over-expressed genes between old mice treated with the three-compound combination (5-azacytidine, tranylcypromine and JNK-IN-8), and old mice treated with the vehicle. The level of significance for considering a gene set enrichment was FDR ≤ 0.05, and NAB/expected NAB > 1, which reflects that the differentially expressed genes are over-represented in the reference set.
